# Cold Atmospheric Plasma Boosts Virus Multiplication via EGFR(Tyr1068) Phosphorylation-Mediated Control on Cell Mitophagy

**DOI:** 10.7150/ijbs.71983

**Published:** 2022-05-09

**Authors:** Peiyu Han, Li Shen, Nan Nan, Renwu Zhou, Tianci Li, Xiaofeng Dai

**Affiliations:** 1 Wuxi School of Medicine, Jiangnan University, Wuxi 214122, China.; 2 School of Chemical and Biomolecular Engineering, University of Sydney, Sydney, 2008, Australia.; 3 CAPsoul Medical Biotechnology Company, Ltd, Beijing, 100000, China.

**Keywords:** Cold atmospheric plasma, EGFR, Mitophagy, Cell cycle, Vaccine, Virus

## Abstract

**Objectives:** Vaccination still remains as the most effective approach for preventing infectious diseases such as those caused by virus infection, with cell-based vaccine manufacturing being one flexible solution regarding the spectrum of infectious disorders it can prevent. Rapid cell-based virus propagation can enable high yield of vaccines against viral diseases that may offer critical values in the industry when handling emergent situations such as the ongoing viral disease pandemic.

**Methods:** Through investigating the phenomenon and biological mechanism underlying redox-triggered cell survival towards enhanced viral particle production, this study explores novel strategies for improved yield of viral particles at a reduced cost to meet the increasing demand on cell-based vaccine manufacturing against viral diseases.

**Results:** We found in this study that cold atmospheric plasma (CAP), composed of multiple reactive oxygen and nitrogen species including H_2_O_2_, could effectively enhance virus replication via triggering cell mitophagy that was dynamically modulated by the p-EGFR(Tyr1068)/p-Drp1(Ser616) axis using IBRV and MDBK as the virus and cell models, respectively; and removing H_2_O_2_ can further enhance virus yield via releasing cells from excessive G_0_/G_1_ cell cycle arrest. The observed efficacy of CAP was extended to other viruses such as CDV and CPV.

**Conclusion:** This study provides experimental evidences supporting the use of CAP as a modulator of cell survival including mitophagy and mitochondria dynamics, and makes CAP an interesting and promising tool for enhancing the yield of viral vaccines if translated into the industry.

## Introduction

Vaccination is an effective approach for infectious disease prevention and possibly the only solution for eradicating viral diseases that have low virulence but rapid transmission and with the potential of eventually evolving into symbiosis with human. Cell-based vaccine manufacturing that utilizes cultured mammalian cells as the virus production factory represents a flexible approach for viral vaccine production regarding the yield and spectrum of viral diseases it can control 1. Rapid vaccine production can deliver critical benefits especially under urgent situations such as the outbreak of Coronavirus Disease 2019 (COVID-19) epidemic 2. Enhancing virus titer is a practical strategy to meet the large and increasing demand on cell-based vaccine manufacturing against viral diseases.

Cold atmospheric plasma (CAP), belonging to the fourth state of matter, has been shown capable of preventing COVID-19 3, deactivating viruses, removing pathogens such as bacteria and fungi 4, and selectively killing cancer cells via inducing, e.g., cell cycle arrest 5, senescence 6, autophagy 7, apoptosis 8, ferroptosis 9, 10, ICD 11, and necrosis 12 in a dose-dependent fashion 13. It is composed of long-lived and short-lived reactive oxygen and nitrogen species, the chemical properties of which have been intensively studied 14-19. We had previously shown that CAP could hijack host signaling towards enhanced virus production under controlled dosing and following specified protocol 7, 20. It is thus possible that CAP can function as a tool to boost virus multiplication that ultimately leads to rapid vaccine production for viral disease control.

Motivated as such, we explored the efficacy of CAP in triggering increased virus titration and the underlying biological mechanism with the focus laid on cell survival including selective autophagy and cell cycle arrest. Using infectious bovine rhinotrachieitis virus (IBRV) and Madin-Darby Bovine Kidney (MDBK) cells as the virus and cell models, respectively, we found that CAP could boost virus propagation through enhancing cell mitophagy that was dynamically modulated by the activity and location of phosphorylated EGFR (Tyr1068). Interestingly, removing H_2_O_2_ from CAP-activated medium (PAM) further enhanced virus titer via releasing cells from H_2_O_2_-triggered halt at the G_0_/G_1_ stage. Similar effect of CAP on boosting virus titer was observed for other viruses such as canine parvovirus (CPV) and canine distemper virus (CDV). Our study makes CAP an effective approach enabling rapid viral vaccine manufacturing and, also a potential dose-dependent tool for modulating cell mitophagy and mitochondria dynamics towards desirable cell outcomes for other research and clinical purposes.

## Materials and methods

### Home-made CAP ejection device and parameter configuration

The home-made CAP ejection device is composed of a power controller, a helium (He) gas cylinder, a rotor flow meter, and a CAP jet (**Supplementary Figure 1**). The peak-to-peak voltage applied to the electrode, the sinusoidal wave frequency, the He flow rate, and the distance from the CAP jet to the medium surface were set to 0.96-1.24 kV, 10 kHz, 1 L/min, and 13 mm, respectively. Plasma activated medium (PAM) was prepared by exposing 1 mL medium to CAP for 2 or 4 min for each well in a 24-well plate.

### Cells and viruses

IBRV (NCBI TaxId: 79889) was purchased from the Type Culture Collection of the Chinese Academy of Sciences, Shanghai, China and propagated using MDBK cells. The MDBK cell line was purchased from Cell Bank of the Chinese Academy of Sciences Cell Bank and cultured in RPMI medium containing 10% fetal bovine serum (FBS) and 1% penicillin-streptomycin.

### Virus infection and CAP treatment

Cells were grown to 80%-90% confluence prior to incubation with different doses of PAM for 1 h. Cells were infected with viruses for 1 h. The medium was refreshed, and cells were cocultured with viruses for additional 46 h.

### Tissue culture infective dose

Tissue culture infective dose (TCID50) was measured as the virus dilution fold when 50% cells were infected. Cells were plated in a 96-well plate. Inoculating cells with a 10-gradient sequentially diluted solutions of viral fluid at a step-size of 10 folds, and monitoring cells for 5 to 7 days until the occurrence of cytopathic effect (CPE) in 50% cells. TCID50 was calculated following previously recommended protocol 21.

### qRT-PCR

Basic qRT-PCR protocol: 4 μL cDNA samples, 10 μL 2×UltraSYBR MixTure, 1 μL forward and 1 μL backward primers, 4 μL ddH_2_O were mixed and centrifuged before running the qRT-PCR program (pre-denature at 95 °C for 10 min; 95 °C for 10 s, 60 °C for 1 min, 72 °C for 20 s, for 40 cycles) in Roche LightCycler 480 RT-PCR. Each sample had 3 replicates.

For virus detection, supernatants were collected and boiled following the protocol of UltraSYBR MixTure Kit (Catalog Number: CW0957M, Cwbio Co. Ltd., China) prior to the aforementioned basic q-PCR protocol. For EGFR mRNA quantification, intracellular mRNA was extracted followed by reverse transcription into cDNA prior to the aforementioned basic q-PCR protocol.

Primers for IBRV detection and EGFR quantification were listed in **Supplementary Table 1**.

### Western blot

Cells were grown in 6-well plates, washed twice with PBS, lysed on ice for 20 min using RIPA lysis buffer containing protease inhibitor, and centrifuged at 12,000 g for 20 min before collecting the supernatants. Protein concentration was estimated using BCA analysis kit (Catalog Number: P0012 Beyotime Biotechnology, China). The protein per lane (20 μg) was resolved by SDS-PAGE and transferred to a PVDF membrane. After blocking with 5% skim milk powder in TBS and Tween-20 buffer, the membrane was incubated overnight at 4 °C with a suitable primary antibody followed by incubation with the secondary antibody for 1 h at the room temperature. After TBST cleaning, the membrane was detected by Bio-Rad Gel Doc XR. Details on antibodies used in this study were summarized in **Supplementary Table 2**.

### Immunofluorescence

Cells were fixed with cell fixature and permeabilized with 1% Triton X-100. Cells were blocked in 1% bovine serum albumin (BSA) followed by incubation with the primary antibodies for 8 h. Cells were incubated with the secondary antibody and imaged using laser-scanning confocal microscope Zeiss Axio Imager Z2 (Zeiss, Germany).

For mitochondria morphology imaging, cells were incubated with 100 nM MitoTracker (Catalog Number: M7512 Thermo Scientific, USA) for 30 min at 37 °C prior to the performance of the aforementioned protocol.

### Mutation of EGFR(Tyr1068) to EGFR(Phe1068)

The pRP[CRISPR]-EGFP/Puro-hCas9-U6>{EGFR[gRNA]} and Single-stranded Oligo Donor (ssODN) (CAGGGATCCTGCATGGGATGGTGCTTTGCTGATTACTTCACCTCTGATTTCTTTCCACTTTCAGAGTTCATTAATCAGTCCGTTCCCAAAAGGCCCGCTGGCTCTGTGCAGAATCCTGTCTATCACAATCAGCCTCT) were purchased from Vector Builder. The ssODN-EGFR was ethanol precipitated and re-suspended (100 μM) in 10 mM Tris-HCl (pH=7.5). Oligos of EGFR(Tyr1068) (not including the signal peptide of 24 Aa at N-terminal) were diluted to 1 μM prior to transfection. When MDBK cells grew to 60% -70% confluence in 6-well plates, 1 μM ssODN, 1 μg pRP[CRISPR]-EGFP/Puro-hCas9-U6 and 6 μL lipofectamine 3000 were added followed by incubation for 8 h.

### Cell cycle assay

Cells were grown in 6-well plates, washed using PBS and digested with EDTA-free trypsin. Cells were centrifuged at 1000 rpm for 5 min to retain the pellets. Cell pellets were washed with 500 μL PBS, centrifuged at 1000 rpm/min for 5 min, and the supernatant was removed to retain the pellet. Cell pellets were re-suspended in 70% ethanol and placed in a refrigerator at 4 °C overnight for fixation. Fixed cells were centrifuged at 1000 rpm for 5 min to remove the supernatant, and cell pellets were suspended in 500 μL PBS. 5 μL propidium iodide (Catalog Number: ST511, Beyotime Biotechnology, China) was added and mixed with cells on ice for 30 min. Cell cycle detection was performed using a BD Accuri C6 flow cytometer, and data analysis was performed using Flowjo software.

### ROS detection assay

DCFH-DA (Catalog Number: S0033S, Beyotime Biotechnology, China) was diluted with serum-free culture medium at a ratio of 1:1000 to make the final concentration 10 mM/L. Cell culture medium was removed followed by addition of DCFH-DA in an appropriate volume. Cells were incubated in a 37 °C cell incubator for 20 min. Cells were washed 3 times using serum-free cell culture medium to fully remove the rest of DCFH-DA. Cells were collected by trypsin without adding EDTA. Real-time fluorescence intensity detection was performed using Zeiss Axio Imager Z2 (Zeiss, Germany) before and after the signal stimulation using 488 nm excitation wavelength and 525 nm emission wavelength.

### Scavenger assay

Mannitol (200 mM, Catalog Number: M8140, Solarbio, China), sodium pyruvate (10 mM, Catalog Number: SP0100, Solarbio, China), uric acid (100 μM, Catalog Number: IU0150, Solarbio, China), tiron (20 mM, Catalog Number: T104954, Aladdin, China), hemoglobin (20 μM, Catalog Number: H8020, Solarbio, China), and monopotassium phosphate (1 mM, Catalog Number: P7392, Solarbio, China) were used to quench hydroxyl radical (OH·), hydrogen peroxide (H_2_O_2_), ozone (O_3_), superoxide anion (O_2_^·-^), nitric oxide (NO·), and electron (e^-^), respectively.

After treating cells with CAP for 2 or 4 min followed by incubation for 1 h, the quencher of each CAP component was added, separately. Viruses were added to cells and incubated for 1 h. The medium was refreshed, and cells were cocultured with viruses for additional 46 h prior to subsequent analyses.

### Optical emission spectroscopy (OES)

The optical emission spectrum, in the gas phase, of CAP ejected from our home-made He-based device (**Supplementary Figure 1**) was detected using a spectrometer (Andor Shamrock SR-500i-A-R, England), where the optical probe was placed at a distance of approximately 10 mm from the center of the ejection device.

### Gene expression determination

Gene expression, represented as FPKM (Fragments Per Kilo Per Million reads) 22, was computed using Htseq software (V 0.6.1) following **Equation 1**.


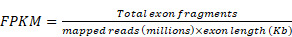

(1)

The mapped reads represent how much percent of all reads are mapped to a gene divided by the length of the gene. FPKM normalization normalizes both the sequencing depth and the gene length to make the expression of genes at different lengths and different sequencing depths comparable. In general, FPKM value 0.1 or 1 is considered as the threshold to determine whether a gene is expressed.

### RNA-Sequencing correlation analysis

The correlation of gene expression levels between samples is an important index to test the reliability of the experiments. Pearson correlation was conducted among samples in a pair-wise fashion, with R^2^>0.8 being considered highly correlated.

### Principle component analysis

Principle component analysis (PCA) was conducted using R packages 'gmodels', 'ggplot2', 'ggrepel'.

### Clustering analysis

A hierarchical clustering analysis was performed based on the FPKM values of all genes in different samples, where 'euclidean' was used as the distance and 'complete linkage' was set as the linkage approach.

### Identification of differentially expressed genes

Differentially expressed genes were identified using the 'DESeq2' bioconductor package, where genes differentially expressed over 2 folds between the compared groups were considered as differentially expressed, and those with adjusted p value ≤ 0.05 and FDR ≤ 0.05 were selected.

### Gene ontology analysis

Gene ontology (GO) analysis was conducted using GOseq 23 that is based on Wallenius non-central hyper-geometric distribution. Threshold used for enrichment filtering was set as p ≤ 0.05 for over represented genes.

### KEGG pathway analysis

KEGG (Kyoto Encyclopedia of Genes and Genomes) 24 analysis was conducted using the 'clusterProfiler' package 25 from R. The analysis was conducted using hypergeometric test where KEGG pathway was considered as the unit following **Equation 2**.


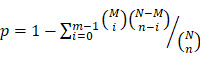

(2)

In Equation 2, 'N' is the number of genes annotated in the pathways among all genes, 'n' is the number of differentially expressed genes among 'N', 'M' is the number of genes annotated to the specific pathway among all genes, and 'm' is the number genes annotated to the specific pathway among differentially expressed genes. Threshold for enrichment significance was set as p value ≤ 0.05.

## Results

### CAP enhances IBRV titration through ROS-triggered selective autophagy

**Figure 1:**
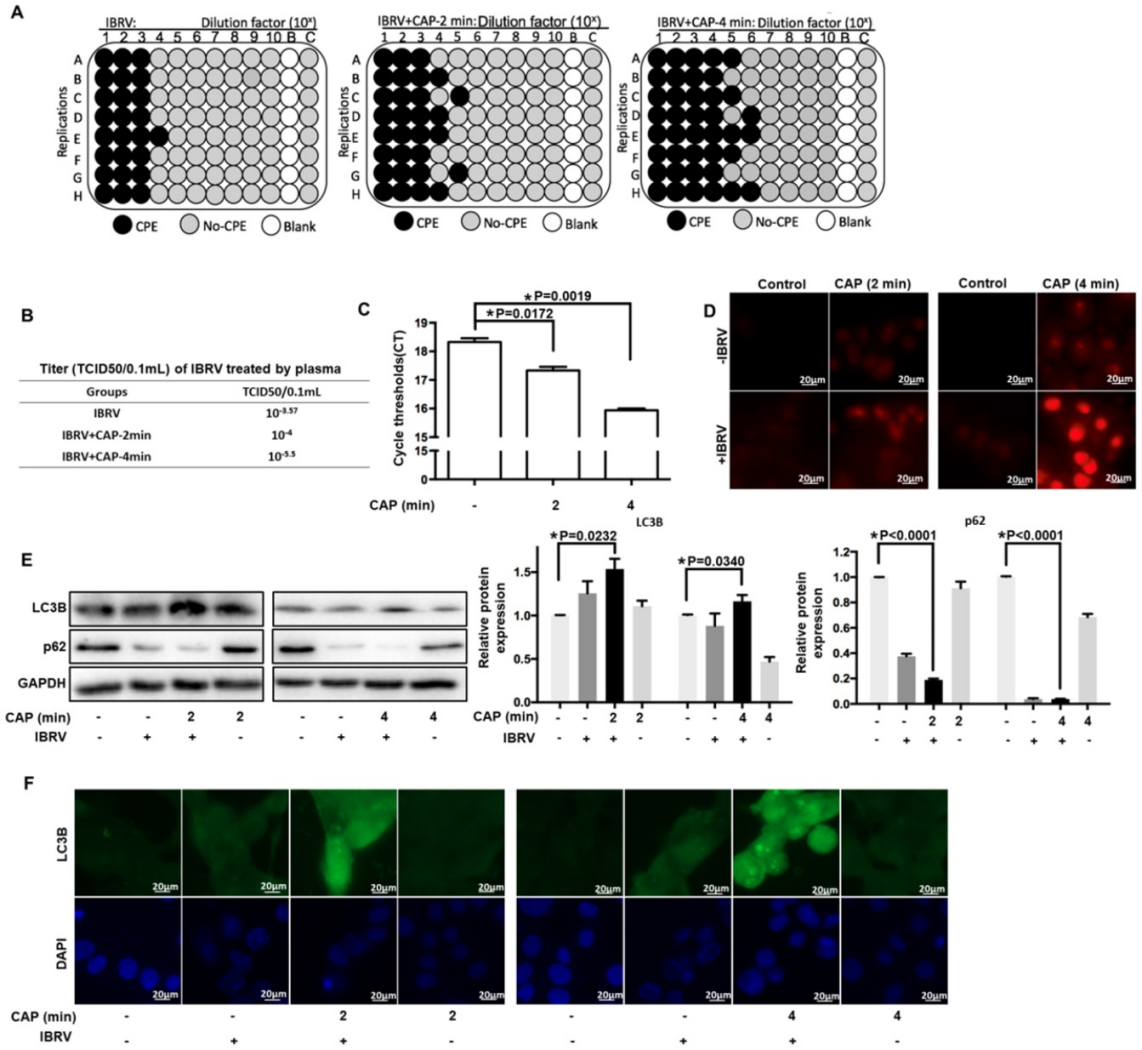
** IBRV titer and autophagy on IBRV infection and/or CAP exposure at different doses in MDBK cells. (A)** Proportion of MDBK cells undergoing CPE after joint CAP exposure and IBRV infection in MDBK cells. IBRV titer in response to CAP 2 min and 4 min exposure as measured using **(B)** TCID50 and **(C)** qRT-PCR in MDBK cells. **(D)** Cellular ROS level after IBRV infection or CAP exposure in MDBK cells. **(E)** Western blots and their quantifications on key genes involved in autophagy (LC3B, p62) in MDBK cells. **(F)** Immunofluorescence imaging showing synergies between CAP exposure and IBRV infection on increasing LC3B cellular level in MDBK cells.

Through activating the cell culture medium (**Supplementary Figure 1**), CAP could substantially enhance the number of cells undergoing CPE after IBRV infection (**Figure 1A**) and effectively increase IBRV titer from 10^-2.57^/mL to 10^-3^/mL on 2 min CAP exposure and to 10^-4.5^/mL on 4 min exposure (**Figure 1B**). The qRT-PCR also revealed reduced PCR cycles required for IBRV detection on 2 min and 4 min CAP exposure (**Figure 1C**).

CAP is featured by dose-dependent cellular redox level modulation 8, 13, 26, 27. Substantially enhanced level of ROS was observed on 4-min CAP exposure as compared with 2-min CAP exposure in IBRV-infected MDBK cells (**Figure 1D**).

Selective autophagy is one important process governing virus replication 20 and constitutes to an important part of cell stress responses including redox stress in mammalian cells 28. P62 is the best characterized selective autophagy substrate in mammals that localizes at the autophagosome formation site 29, and LC3B is responsible for selective degradation of p62 through selective autophagy 30, both of which are typical markers of selective autophagy 31. We observed considerably elevated LC3B and substantially reduced p62 expression on joint CAP exposure and IBRV infection (**Figure 1E**). The fluorescence imaging signal of LC3B in cells receiving both CAP exposure and IBRV infection was considerably higher than that in cells treated with CAP or IBRV alone (**Figure 1F**). These results collectively suggested the accompany of selective autophagy in CAP-triggered increase on virus titer.

### EGFR(Tyr1068) phosphorylation plays a central role in increasing IBRV titer on CAP exposure

**Figure 2:**
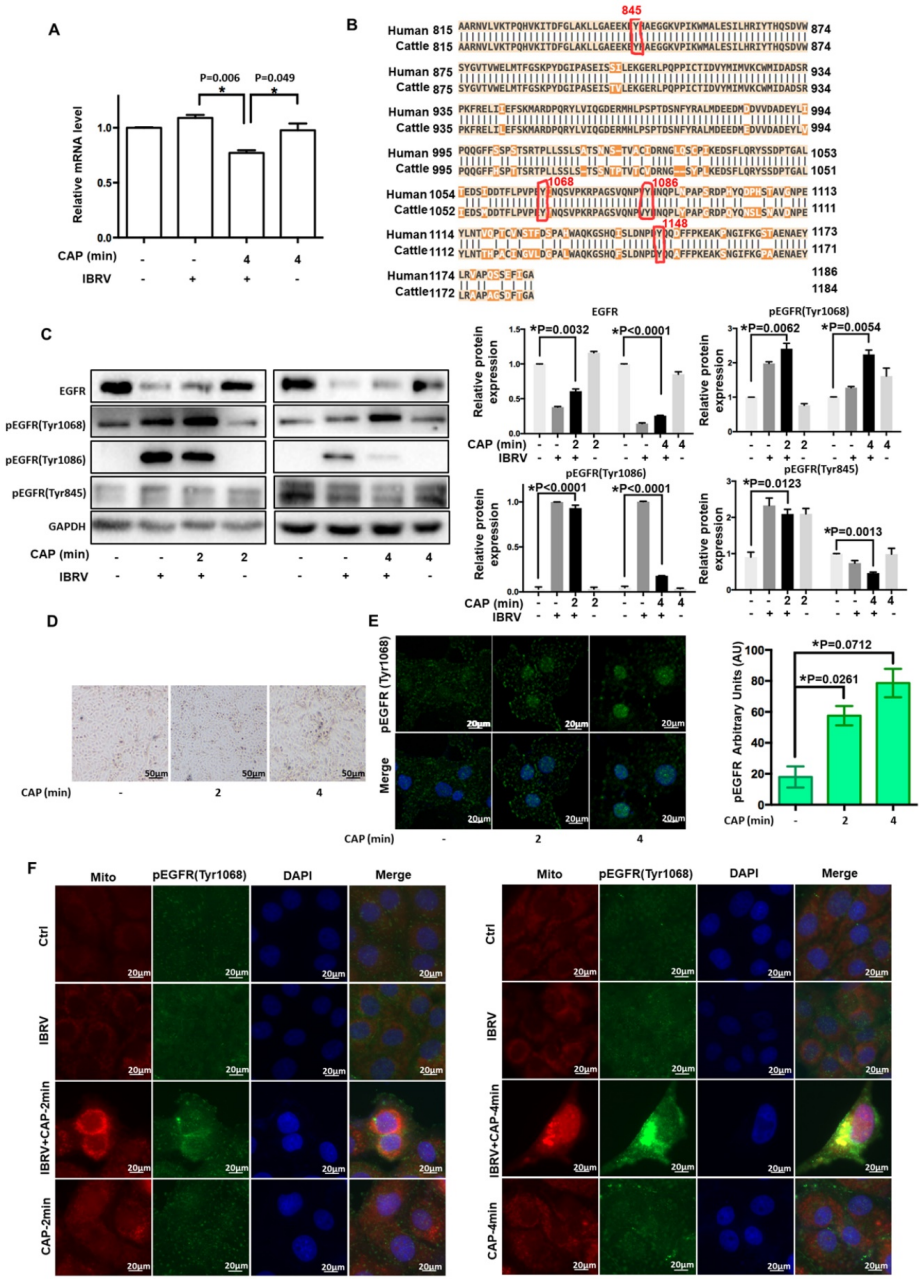
** Expression and activity of EGFR and different EGFR phosphorylation sites on IBRV infection and/or CAP exposure at different doses. (A)** EGFR mRNA level on 4-min CAP exposure, IBRV infection and their combinatorial treatment. **(B)** Conservativity of EGFR phosphorylation sites Tyr1068, Tyr1148, Tyr1086, Tyr845 between human and bovine cells. **(C)** EGFR protein expression and its phosphorylation levels at Tyr1068, Tyr1148, Tyr1086, Tyr845, as well as their quantifications. **(D)** Imaging of cells in response to CAP exposure at different doses. **(E)** Immunofluorescence imaging of EGFR (Tyr1068) expression on CAP exposure at different doses. **(F)** Immunofluorescence imaging showing colocalization of mitochondria and EGFR (Tyr1068) on combined treatment of IBRV infection and CAP exposure at different doses.

Various evidences have supported the roles of EGFR in ROS-induced cellular response 32-37. EGFR level was considerably reduced when cells were exposed to IBRV infection and 4-min CAP treatment followed by 46 h culturing (p<0.001, **Figure 2A**). EGFR phosphorylation sites (Tyr1068, Tyr1148, Tyr1086, Tyr845) are highly conserved between human and bovine protein sequences (**Figure 2B**), enabling the use of human rather than bovine antibodies in the following experiments here.

For all the four phosphorylation sites examined, p-EGFR (Tyr1068) was synergistically enhanced by IBRV infection and CAP exposure (**Figure 2C**). With the increase of CAP dose (from 2 min to 4 min) that did not trigger cell death (**Figure 2D**), p-EGFR(Tyr1068) started to accumulate in cell nucleus (**Figure 2E**). IJoint exposure of cells to CAP and IBRV infection substantially enhanced p-EGFR(Tyr1068) level that was co-localized with mitochondria (**Figure 2F**), associating observed selective autophagy to mitophagy.

### EGFR(Tyr1068) phosphorylation enhances mitophagy on CAP exposure and IBRV infection through interacting with phosphorylated Drp1(Ser616)

**Figure 3:**
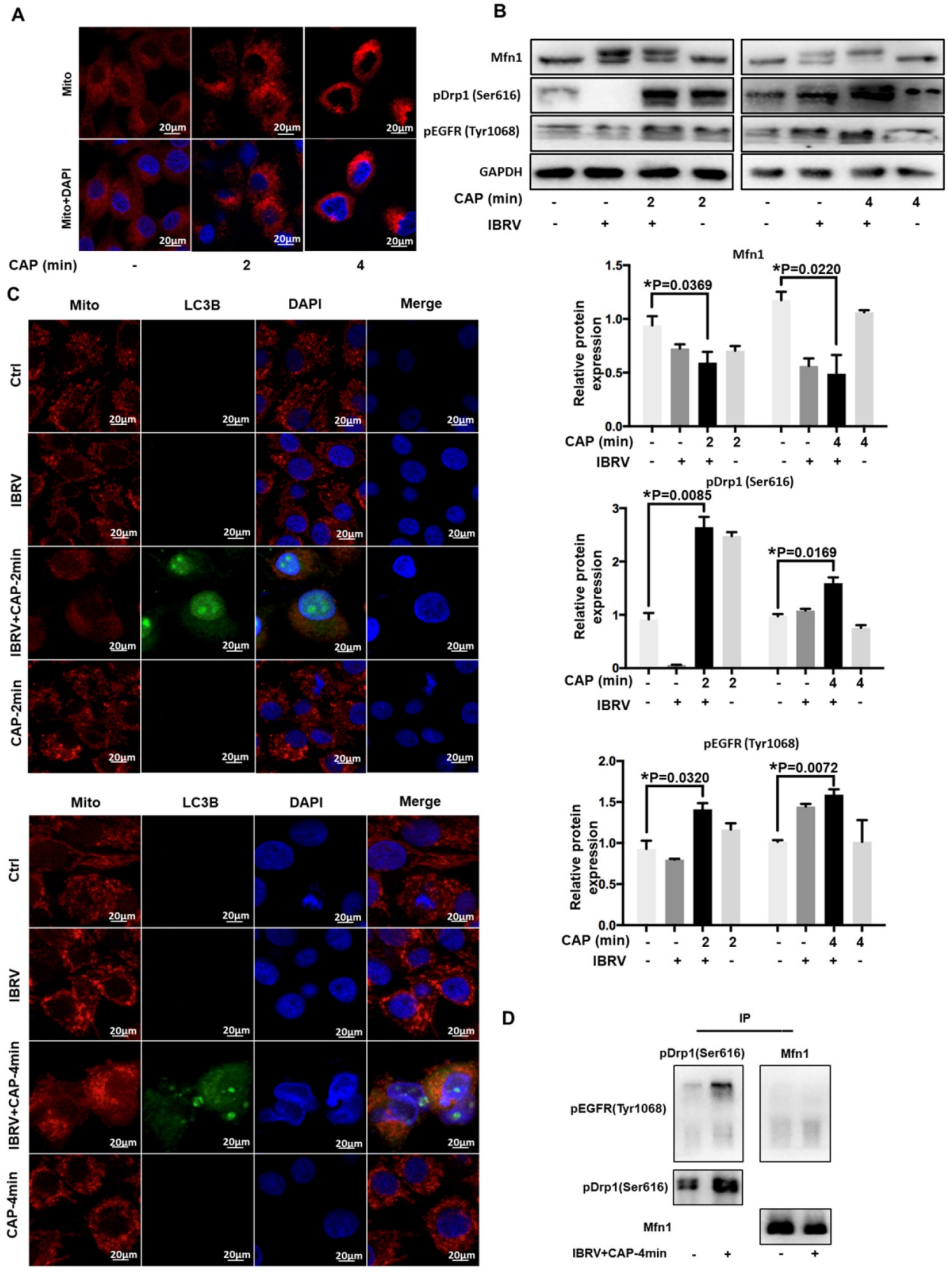
** CAP triggers mitophagy via direct interactions between EGFR(Tyr1068) and Drp1(Ser616). (A)** Morphological alteration of mitochondria on exposure to different doses of CAP. **(B)** Mitochondrial dynamic-related protein expression on IBRV infection, CAP exposure, their joint treatment, and their quantifications. **(C)** Mitochondria and LC3B colocalization on IBRV infection, CAP exposure and their joint treatment. **(D)** Immunoprecipitation of Drp1(Ser616) and EGFR(Tyr1068).

We next examined the role of p-EGFR(Tyr1068) in CAP-triggered mitophagy after IBRV infection. Mitochondria accumulated to the nucleus surrounding area, and the signal was intensified with the increase of CAP treatment duration (**Figure 3A**), implicating the occurrence of mitophagy. Mitofusin 1 (Mfn1) and dynamin-related protein 1 (Drp1) coordinate the processes of mitochondrial fusion and fission, which are canonical markers of mitophagy 38, 39. On joint exposure to IBRV infection and CAP treatment, p-EGFR(Tyr1068) and p-Drp1(Ser616) expression were substantially increased, whereas Mfn1 level was reduced (**Figure 3B**), suggesting the occurrence of cell mitophagy and involvement of p-EGFR(Tyr1068) in cells' response to both CAP exposure and IBRV infection. LC3B was co-localized with mitochondria and the signal was intensified on joint exposure to CAP and IBRV infection (**Figure 3C**), confirming the presence of mitophagy. Co-immunoprecipitation suggested physical interactions of p-EGFR(Tyr1068) with p-Drp1(Ser616) but not with Mfn1 on joint exposure to IBRV infection and CAP treatment (**Figure 3D**), indicating that the enhanced cell fission was mediated via the p-EGFR(Tyr1068)/p-Drp1(Ser616) axis.

### EGFR(Tyr1068) mutation reduces virus titer and mitophagy on CAP exposure after IBRV infection

**Figure 4:**
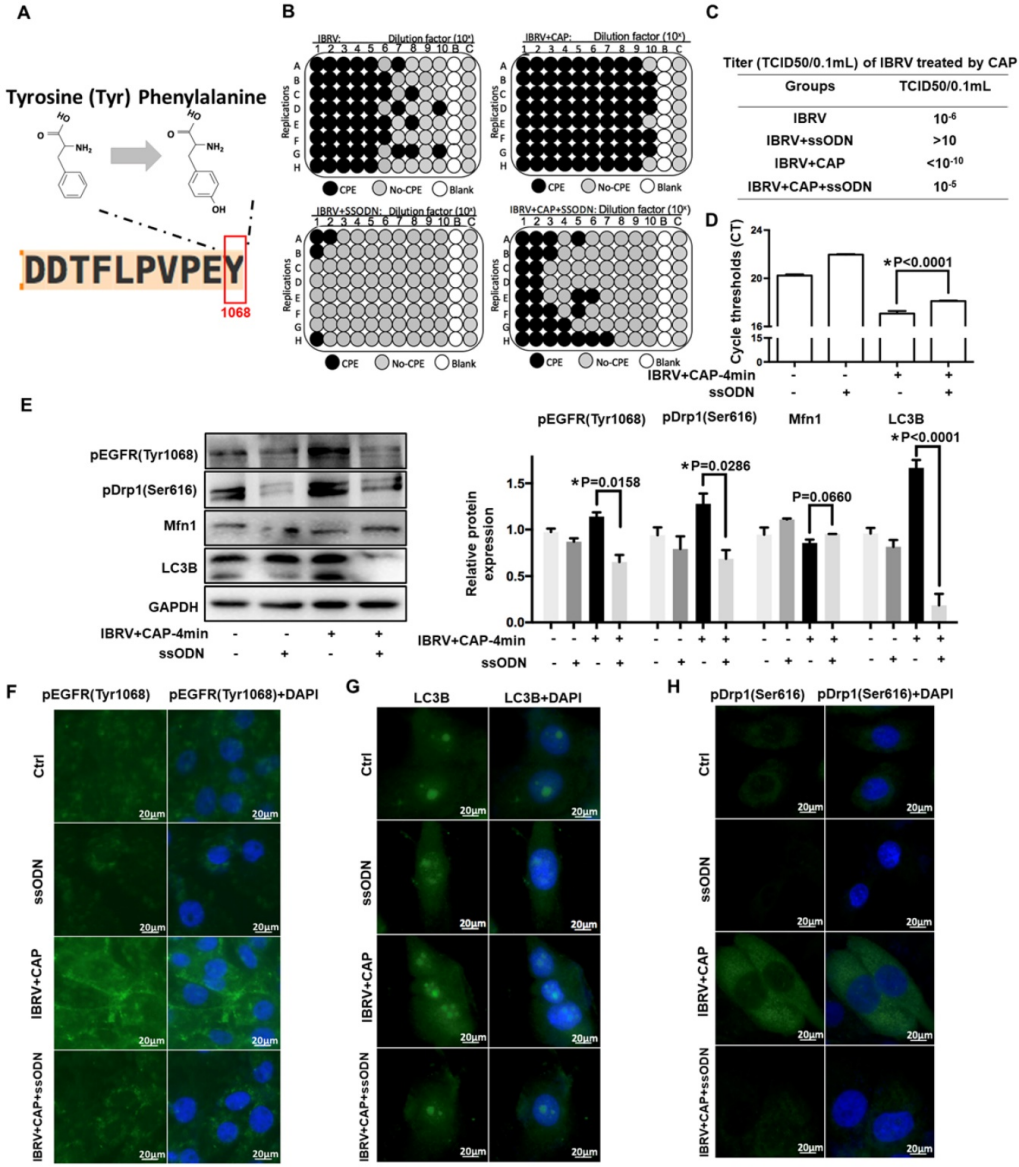
** IBRV titer and autophagy on IBRV infection and/or CAP exposure in cells deficient of EGFR(Tyr1068) through ssDNA-mediated point mutation. (A)** Point mutation of EGFR(Tyr1068) from Tyrosine to Phenylalanine used in this study. **(B)** Proportion of MDBK cells undergoing CPE after joint CAP exposure and IBRV infection in EGFR(Tyr1068)-mutated MDBK cells. IBRV titer in response to CAP 2 min and 4 min exposure as measured using **(C)** TCID50 and **(D)** qRT-PCR in EGFR(Tyr1068)-mutated MDBK cells. **(E)** Western blots and their quantifications on EGFR(Tyr1068), key genes involved in autophagy (LC3B, p62) and mitophagy (Drp1, Mfn1) in EGFR(Tyr1068)-mutated MDBK cells. Immunofluorescence imaging of **(F)** EGFR(Tyr1068), **(G)** LC3B and **(H)** Drp1(Ser616) in EGFR(Tyr1068)-mutated MDBK cells. 4-min CAP exposure was used in these assays. Controls used in this assay were cells transfected with an empty vector to remove potential off-target effects.

To consolidate the role of p-EGFR(Tyr1068) in CAP-triggered increase on virus titer and mitophagy after IBRV infection, we generated an EGFR(Tyr1068) mutant plasmid where tyrosine (Tyr) at the 1068 site was mutated to phenylalanine (Phe) (**Figure 4A**). The p-EGFR(Tyr1068) level was successfully reduced in cells transfected with the mutant plasmid (**Figure 4E**, **4F**). Cells transfected with the mutant plasmid produced considerably less CPE after IBRV infection with and without CAP exposure (**Figure 4B**). Accordingly, virus titer reduced substantially (**Figure 4C**) and the Ct values from the qRT-PCR assay were significantly enhanced in EGFR(Tyr1068) mutant cells (**Figure 4D**). Each primary marker used for selective autophagy and mitophagy characterization (i.e., LC3B, p-Drp1(Ser616), Mfn1) exhibited a relapsed pattern in EGFR(Tyr1068) mutant cells as compared with the wild type under the same treatment (**Figure 4E, 4G, 4H**).

### The effects of CAP components on virus titer

**Figure 5:**
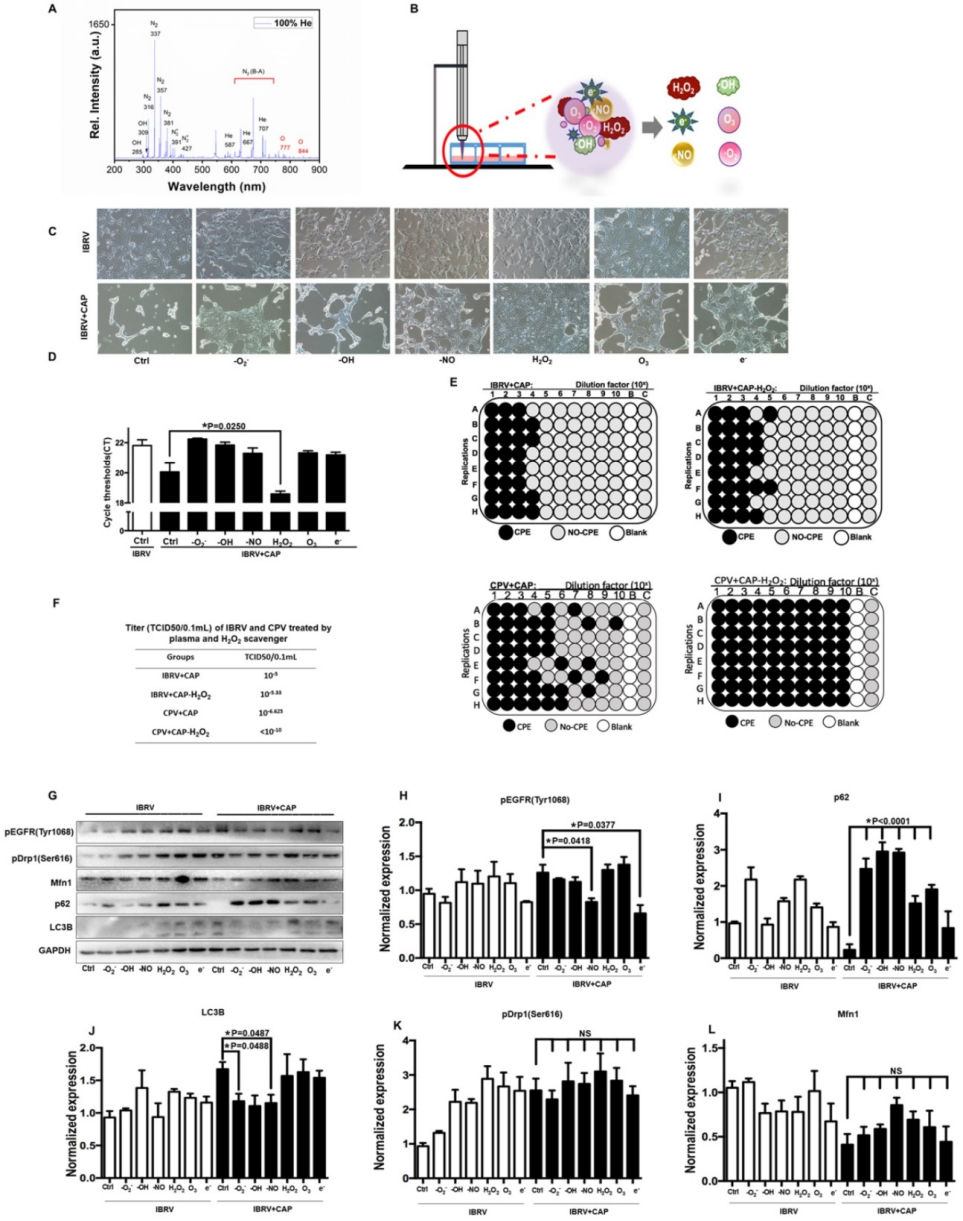
** Effect of each CAP primary component on virus infected MDBK cells. (A)** Optical emission spectroscopy of CAP ejected from the home-made device ( Supplementary Figure 1). **(B)** Schematic diagram on CAP-activated medium preparation and its primary components. **(C)** Morphology, **(D)** q-RT-PCR cycle thresholds (Ct) of IBRV infected MDBK cells with and without CAP exposure after removing each primary component. **(E)** Plate configuration and **(F)** statistics showing TCID50 of IBRV or CPV infected MDBK cells on CAP exposure with and without removing H_2_O_2_. **(G)** Western blots and quantification of **(H)** EGFR(Tyr1068), **(I)** p62, **(J)** LC3B, **(K)** Drp1(Ser616), **(L)** Mfn1 of IBRV infected MDBK cells with and without CAP exposure after removing each primary component.

CAP ejected from our home-made He-based device (**Supplementary Figure 1**) was primarily composed of OH, N_2_, N_2_^+^ and O in the gas phase (**Figure 5A**), which became H_2_O_2_, O_3_, •O_2_^-^, •OH, •NO, e^-^ in the liquid form 40 (**Figure 5B**). By removing/quenching H_2_O_2_ from PAM, cell amount (**Figure 5C**) and virus yield (**Figure 5D**) were substantially increased after PAM treatment. We next confirmed the roles of H_2_O_2_ in decreasing virus titer using TCID50, i.e., it dropped from 10^-5^ to 10^-5.33^ in IBRV-infected cells and from 10^-6.625^ to <10^-10^ in CPV-infected cells on CAP exposure (**Figure 5E, 5F**).

The p-EGFR(Tyr1068) mediated virus titer increase was primarily triggered via •NO and e^-^ given the dramatic decrease on p-EGFR(Tyr1068) expression after removing each of these species (**Figure 5G, 5H**). The effect of H_2_O_2_ on virus titer was in-dependent of p-EGFR(Tyr1068) signaling as no visible alteration was observed regarding p-EGFR(Tyr1068) expression when H_2_O_2_ was removed (**Figure 5G, 5H**), suggestive of the involvement of additional mechanisms in virus titer control. Autophagy as assessed via p62 and LC3B expression here was primarily affected by •O_2_^-^, •OH, •NO that, once being removed separately, showed opposite profiles on CAP exposure (**Figure 5I, 5J**). Mitochondria fission through p-Drp1(Ser616) and fusion via Mfn1 were not primarily affected by any of these components as the fluctuations were not dramatic by adding any of these reactive species quenchers (**Figure 5G, 5K, 5L**), suggestive of the combinatorial role of these species in triggering cell mitophagy.

### Transcriptome sequencing predicts the role of cell cycle arrest in H_2_O_2_-triggered virus titer decrease and consolidates EGFR-mediated virus titer increase on CAP exposure

**Figure 6:**
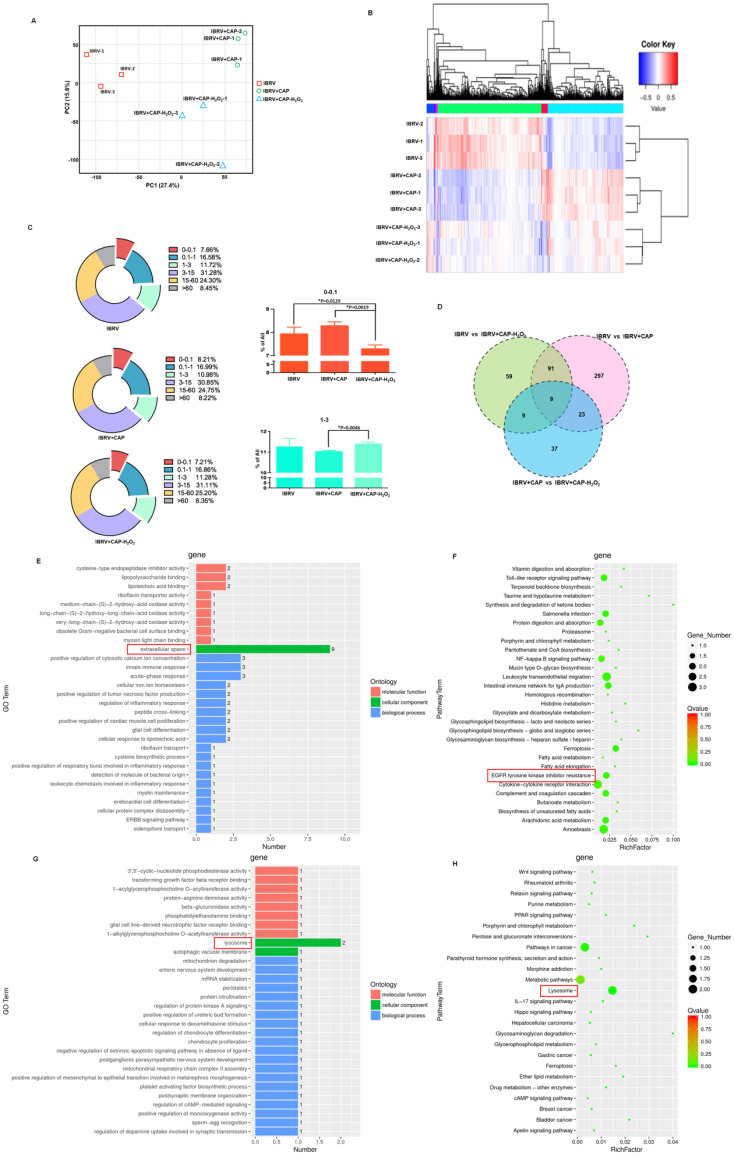
** Effects of CAP and H_2_O_2_ on virus multiplication and intracellular environment revealed from transcriptome sequencing. (A) Principal** component analysis and** (B)** heatmap showing relationships among sample groups.** (C)** Gene counts at different expression levels among sample groups.** (D)** Venn diagram of genes differentially expressed between sample groups. **(E)** Gene ontology and** (F)** KEGG pathways enriched with genes differentially expressed in pair-wise comparisons of 'IBRV vs IBRV+CAP' and 'IBRV vs IBRV+CAP-H_2_O_2_' but not in 'IBRV+CAP vs IBRV+CAP-H_2_O_2_'. **(G)** Gene ontology and** (H)** KEGG pathways enriched with genes differentially expressed in the pair-wise comparison of 'IBRV+CAP vs IBRV+CAP-H_2_O_2_' but not in the other two pairs. There are 3 sample groups: IBRV, IBRV+CAP, IBRV+CAP-H_2_O_2_. Each sample group contains 3 replicates that are denoted as '_1', '_2', '_3'. IBRV: IBRV-infected MDBK cells infected with IBRV, IBRV+CAP: IBRV-infected MDBK cells treated with CAP; IBRV+CAP-H_2_O_2_: IBRV-infected MDBK cells treated with CAP and H_2_O_2_ scavenger.

We next conducted transcriptome sequencing to explore the mechanism of H_2_O_2_ in decreasing virus titer and gain a broad view on CAP-triggered cell response. Gene expression did not considerably vary among sample groups (**Supplementary Figure 2A**), and those of samples within the same group were highly correlated (**Supplementary Figure 2B**). Samples of the same group were clustered together from both PCA and clustering analyses (**Figures 6A, 6B**). CAP treatment caused rightward movement of samples along the first principal component (PC1) that explained 27.4% of the total variance; and removing H_2_O_2_ from CAP leftward shifted samples along PC1 and downward along PC2 that explained 15.8% of the total variance (**Figure 6A**). If PC1 reflected the effect of mitophagy, PC2 might represent another cell behavior (namely 'behavior X') that was affected by CAP and, in particular, H_2_O_2_. Additionally, removing H_2_O_2_ decreased the expression of under-expressed (0-0.1) genes (p=0.0129 for IBRV+CAP-H_2_O_2_ vs. IBRV; p=0.0019 for IBRV+CAP-H_2_O_2_ vs. IBRV+CAP) and caused relapsed expression of 1-3 level genes (p=0.0046 for IBRV+CAP-H_2_O_2_ vs. IBRV+CAP) with statistical significance that was oppositely regulated by CAP (**Figure 6C**). These are suggestive of the controversial roles of H_2_O_2_ and CAP in modulating virus titer, as well as the association of these altered genes with the 'behavior X'. Specifically, 420, 168, 78 genes were differentially expressed in the pairwise comparisons 'IBRV vs IBRV+CAP', 'IBRV vs IBRV+CAP-H_2_O_2_' and 'IBRV+CAP vs IBRV+CAP-H_2_O_2_'; and genes simultaneously modulated in all of these three group-wise comparisons showed a consistent regulatory direction in 'IBRV vs IBRV+CAP', 'IBRV vs IBRV+CAP-H_2_O_2_' but an opposite direction in 'IBRV+CAP vs IBRV+CAP-H_2_O_2_' (**Figure 6D, Supplementary Table 3**, **Supplementary Figure 3A**). These results suggested that CAP was the primary contributor to the altered transcriptome profile, and H_2_O_2_ played an opposite role.

Gene ontology (GO) and KEGG pathway analyses revealed that CAP-triggered cell behavior was associated with 'extracellular space' and 'EGFR tyrosine kinase inhibitor resistance', which were the top GO term and pathway, respectively, from both 'IBRV vs IBRV+CAP' and 'IBRV vs IBRV+CAP-H_2_O_2_' group-wise comparisons, but not from the 'IBRV+CAP vs IBRV+CAP-H_2_O_2_' comparison (**Figure 6E, 6F, Supplementary Figure 3B, 3C**). These results are supportive to the mediating role of p-EGFR(Tyr1068) in CAP-triggered mitophagy (**Figure 4**) since the extracellular region of EGFR has been previously proposed as a potential target for anti-EGFR drug discovery 41. The top GO term and KEGG pathway identified from the 'IBRV+CAP vs IBRV+CAP-H_2_O_2_' comparison but not from the other two group-wise comparisons was 'lysosome' (**Figure 6G, 6H**, **Supplementary Figure 3B, 3C**), which is known playing essential roles in autophagy 42. Given the differential regulation of autophagy during cell cycle progression 43 and our knowledge on the roles of H_2_O_2_ in triggering cellular damage and consequently cell cycle arrest under certain concentrations 44, we hypothesized H_2_O_2_-induced cellular 'behavior X' to be 'cell cycle alteration'.

### H_2_O_2_ decreases virus titer through triggering G_0_/G_1_ cell cycle arrest

**Figure 7:**
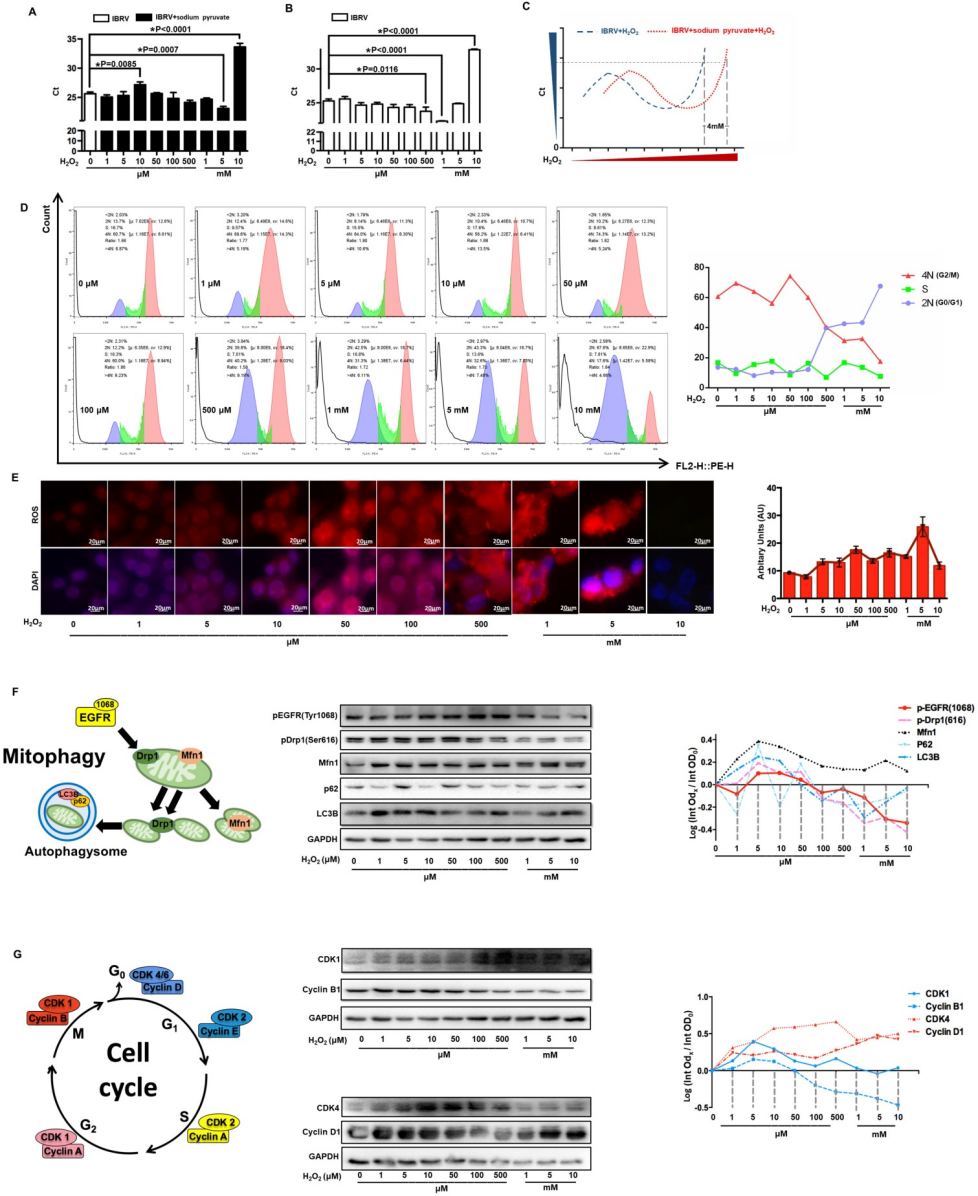
** Effect of H2O2 concentration on virus multiplication and cells. (A)** The qRT-PCR cycle thresholds (Ct) in IBRV-infected MDBK cells supplemented with 10 mM sodium pyruvate (H_2_O_2_ scavenger) and treated with different concentrations of H_2_O_2_. The 1^st^ control (white bar) was not supplemented with sodium pyruvate nor H_2_O_2_. **(B)** Ct values in IBRV-infected MDBK cells treated with different concentrations of H_2_O_2._ **(C)** Schematic illustration on calculating the amount of quenched H_2_O_2_ by 10 mM sodium pyruvate. **(D)** Cell cycle profiles, and **(E)** cellular ROS level of IBRV-infected MDBK cells under treatment of different concentrations of H_2_O_2_. **(F)** Western blots and quantification of p-EGFR(Tyr1068), p-Drp1(Ser616), Mfn1, p62, LC3B in IBRV-infected MDBK cells under different H_2_O_2_ concentrations. Simplified illustration on the relationship between EGFR, autophagy markers (Drp1, Mfn1), and mitophagy markers (p62, LC3B) is also provided. **(G)** Western blots and quantification of proteins critical to the G_2_/M, G_0_/G_1_ cell cycle stages (cyclin B1, CDK1, cyclin D1, CDK4) in IBRV-infected MDBK cells under different H_2_O_2_ concentrations. Simplified illustration on primary cyclins and CDKs controlling the four cell cycle stages is also provided.

We firstly explored CAP-imposed H_2_O_2_ concentration in the medium (or precisely, quenched H_2_O_2_ amount in the scavenger assay) via removing H_2_O_2_ first using 10 mM sodium pyruvate followed by H_2_O_2_ titration, the lowest Ct value of IBRV infected cells was achieved when 5 mM and 1 mM H_2_O_2_ were supplemented, respectively, with and without adding sodium pyruvate (**Figure 7A, 7B**); thus, H_2_O_2_ quenched by 10 mM sodium pyruvate in our experimental setting was approximately 4 mM (5 mM - 1 mM) (**Figure 7C**). On the other hand, virus titer decreased when cellular H_2_O_2_ exceeded 5 mM (**Figure 7B**), suggesting that the intracellular H_2_O_2_ raised in response to virus infection was over 1 mM (5 mM - 4 mM). Either virus-triggered H_2_O_2_ (over 1 mM) or CAP-imposed H_2_O_2_ (approximately 4 mM) led to moderate cell cycle arrest (around 40% G_0_/G_1_ phase) that corresponded to slightly increased virus titer. Both sources of H_2_O_2_, once added together, dramatically decreased virus yield as a result of substantially increased number of cells arrested at the G_0_/G_1_ stage (**Figure 7D**). Cellular ROS increased with H_2_O_2_ dose, and the intensity dramatically dropped at 10 mM (**Figure 7E**), indicative of cell death due to exceeded ROS level. Given these results, together with the known functionality of ROS in triggering cell cycle arrest 45, we assumed that the observed inhibition on virus titer was due to exceeded level of H_2_O_2_.

The levels of p-EGFR(Tyr1068) and p-Drp1(Ser616) were both slightly up-regulated when H_2_O_2_ was low (< 1 mM) and dramatically dropped under high (1-10mM) level of H_2_O_2_ (**Figure 7F, Supplementary Figure 4**), which were supportive of the physical interactions of both phosphorylated proteins (**Figure 3D**) and the opposite role of high dose (1-10mM) of H_2_O_2_ and CAP in triggering p-EGFR(Tyr1068)-mediated cell response. Autophagy markers p62 and LC3B showed a similar pattern with that of p-EGFR and p-Drp1 (**Figure 7F**). Cyclin B1 is primarily distributed in the M phase and serves as the master regulator of the G_2_/M transition 46. CDK1 interacts with 9 different cyclins including cyclin B1 that becomes inactivated at the end of the M phase 47. Both cyclin B1 and CDK1 expression declined with the increased dose of H_2_O_2_ (**Figure 7G, Supplementary Figure 4**), suggestive of a suppressive role of high H_2_O_2_ dose on the M stage. Cyclin D1 is rapidly synthesized in the G1 phase and degraded as cells enter the S phase 48, and CDK4/6 creates complexes with cyclin D1 to mediate cell progression through the G1 phase 49. Cyclin D1 expression showed a positive correlation with H_2_O_2_ dose; CDK4 expression increased with H_2_O_2_ concentration and declined when H_2_O_2_ exceeded 1 mM (**Figure 7G, Supplementary Figure 4**), implicating that cells were accumulated and arrested at the G_0_/G_1_ stage when H_2_O_2_ exceeded 1 mM.

## Discussion

As integrin represents a common receptor mediating virus entry 50 and is linked to intracellular signaling via EGFR tyrosine activation, we focused on the four integrin-dependent EGFR tyrosine phosphorylation residues, i.e., Tyr1068, Tyr1086, Try845, Tyr1173 51 in this study. We excluded Tyr1173 given its primary role in the MAPK cascade and cell proliferation 52 to narrow our focus down to cell selective autophagy/mitophagy. Among the three EGFR tyrosine phosphorylation sites, EGFR(Tyr1068) showed the highest phosphorylation level in response to joint IBRV infection and CAP treatment (**Figure 2C**). The expression profile of p-EGFR(Tyr1068) under varied CAP doses matched well with that of selective autophagy biomarkers (LC3B, p62, **Figure 1E**), in particular mitophagy markers (p-Drp1(Ser616), Mfn1, **Figure 3B**), and virus titer (**Figure 1A-1D**). In addition, p-EGFR(Tyr1068) co-localized with mitochondria (**Figure 2F**). These were, collectively, implicative of the regulatory role of p-EGFR(Tyr1068) in mitophagy. We, therefore, considered p-EGFR(Tyr1068) as the key EGFR phosphorylation site that drove the observed efficacy of CAP in boosting virus titer.

Selective autophagy degrades certain proteins, organelles to maintain cellular homeostasis and, on virus invasion, can be hijacked to recycle cell nutritional resources for virus propagation 20. Elevated LC3B expression and decreased p62 level on CAP exposure after virus infection (**Figure 1E**, **1F**) attributed the promotive role of CAP on virus multiplication to cell selective autophagy. Mitochondria accumulation at the nucleus surrounding region (**Figure 3A**), enhanced Drp1(Ser616) phosphorylation and reduced Mfn1 level (**Figure 3B**, **3C**), providing additional evidence associating CAP-triggered selective autophagy to mitophagy. Mitochondrial fusion, fission, biogenesis and mitophagy constitute mitochondrial dynamics that determines mitochondrial morphology, quality and abundance 53, 54. Activated mitochondrial fusion as represented by elevated Mfn1 38 is protective of mitochondria on pressure. Mitochondrial fission machinery as featured by Drp1 38 actively participates in apoptosis 55 and promotes or inhibits cell death depending on the initial lethal stimulus 56. We observed physical interactions between phosphorylated p-EGFR(Tyr1086) and p-Drp1(Ser616) on IBRV infection and/or CAP exposure (**Figure 3D**), suggesting that mitochondria fission was enhanced due to elevated level of Drp1 that was possibly increased by activated EGFR(Tyr1068). Through introducing the EGFR(Tyr1068) point mutation to native EGFR genes using the CRISPR/Cas9 system (**Figure 4**), we consolidated the role of p-EGFR(Tyr1068) in mediating CAP-induced virus titer increase. To this end, we could preliminarily conclude that CAP promoted IBRV titration via, at least partially, the trigger of cell mitophagy, and this process was driven by p-EGFR(Tyr1068)/p-Drp1(Ser616)-mediated mitochondria fission. We also observed elevated expression of the pro-apoptotic protein caspase 7 (**Supplementary Figure 5**) that was supportive to the pro-apoptotic role of mitochondria fission.

Quenching each component of PAM using chemical scavengers, though being a qualitative approach, has been accepted as a standard measure to examine CAP-liquid interactions and the effect of CAP ingredients on cell behavior, based on the fact that each RONS scavenger has a higher reaction rate (often in an order of magnitude) with its targeted species than with other species 57, 58. Here, we used tiron (20 mM), D-mannitol (200 mM), hemoglobin (20 µM), sodium pyruvate (10 mM), uric acid (100 µM) and monopotassium phosphate (1 mM) to trap ·O_2_^-^, ·OH, ·NO, H_2_O_2_, O_3_ and e^-^, respectively, with the reaction rates being 2.9
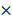
10^9^ mol/Ls, 1
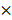
10^9^ mol/Ls, 1
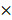
10^8^ mol/Ls, 2.4 mol/Ls, 1.4
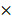
10^6^ mol/Ls 58, 59. Except for H_2_O_2_ whose removal led to a substantial increase on virus titer, eliminating each of the other ingredients unanimously decreased virus multiplication to some extent (**Figure 5D**), suggestive of their indispensable and collective role in promoting virus multiplication.

Exceeded level of H_2_O_2_ (1-10 mM) triggered G_0_/G cell cycle arrest that was associated with recessed cell activities and reduced virus production (**Figure 7**). Thus, cells underwent mitophagy in acute response to CAP-triggered redox stress, leading to the creation of a favorable environment for virus multiplication as a result of shrunken cell biomass usage. However, high H_2_O_2_ dose (1-10 mM) may result in cell cycle G_0_/G_1_ arrest due to the trigger of moderate level of DNA damage signaling (not reaching the threshold of cell apoptosis) and thus counteract virus production. Therefore, a favorable environment for virus multiplication requires enhanced available cellular biomass materials and normal/accelerated cell cycle. Importantly, these results suggested the irreplaceable role of CAP in boosting virus titer that could not be surrogated by H_2_O_2_, an easily produced CAP component, and H_2_O_2_ might function as an opposite regulator of CAP that made its effect on virus titer control reversible.

CAP is a known cocktail of ROS and non-ROS 40. Both CAP exposure and virus infection enhanced cellular redox level 26, 60 (**Figure 1D**), suggesting that the promotive role of CAP in IBRV titer is due to its synergy with virus infection in enhancing cellular ROS level. We observed similar expression profiles between p-EGFR(Tyr1068) and FOXO1 (**Supplementary Figure 5**) on CAP exposure, where FOXO1 is a key measure of cells' anti-oxidative ability 61. This implicated the mediating role of p-EGFR(Tyr1068) in enhancing cellular anti-oxidant ability in response to CAP treatment and/or virus infection.

We additionally found that blocking EGFR(Tyr1068) phosphorylation reduced caspase 3 expression that was elevated on CAP exposure or its joint CAP and IBRV treatments (**Supplementary Figure 6**). This suggested the essential role of p-EGFR(Tyr1068) in regulating cell apoptosis, and that elevated cellular ROS, on CAP exposure, triggered mitophagy and concomitantly tilted cells towards the pro-apoptotic state. This warranted us to keep appropriate CAP dosing to achieve mild yet effective boost of virus titer as over-dosing might kill the host that ultimately led to reduced virus production. We also observed rescued p65 expression in p-EGFR(Tyr1068) mutant cells on CAP or CAP+IBRV treatments (**Supplementary Figure 6**). This implicated that the cellular system was hijacked by viruses for maximum virus production and other cellular behaviors including cell migration (p65 is a key protein controlling cell migration 62) were reduced, whereas p-EGFR(Tyr1068) was the hub controlling these processes.

Though we claimed on the driving role of p-EGFR(Tyr1068) and cell mitophagy in CAP-mediated boost of IBRV propagation, we could not exclude the possible involvement of p-EGFR(Try1086) and p-EGFR(Tyr845) in this process. However, these functionalities were more likely to be associated with cells' natural response to IBRV infection rather than CAP exposure given our results.

Though different viruses may attach and/or trigger cellular signaling using different cell surface receptors beyond EGFR that may limit the generality of the uncovered driving mechanism, the facts that virus infection imposes cells with redox pressure and CAP relies on ROS to deliver its efficacy do not vary. In consistent with this, we observed enhanced titer on CAP exposure when CPV and CDV were used (**Supplementary Figure 7**). Therefore, the efficacy of CAP in boosting virus production may be generalizable to a broad spectrum of, if not all, viruses and hosts that deserves intensive experimental explorations and additional consolidations.

## Conclusion

We report in this study that CAP can enhance IBRV propagation towards increased yield of vaccines against viral diseases through triggering cell selective autophagy and, in particular, mitophagy; this process is driven by the p-EGFR (Tyr1068)/p-Drp1(Ser616) signaling through physical interactions in response to CAP-induced cellular ROS level in a dose-dependent manner (**Supplementary Figure 7A**). High level of H_2_O_2_ (1-10 mM) may decrease virus titer due to enhanced G_0_/G_1_ cell cycle arrest. Thus, controlling H_2_O_2_ below 1 mM through the use of H_2_O_2_ quencher (such as sodium pyruvate) together with CAP may represent a good strategy towards maximal virus production (**Supplementary Figure 7B**). Our results contribute in advancing our understandings on CAP-triggered cellular responses that make CAP a potential modulator on cell survival such as mitophagy and cell cycle arrest. Importantly, this study provides a novel strategy for boosting IBRV multiplication that can be potentially generalized to a broad spectrum of viruses towards enhanced cell-based viral vaccine production in the industry. The generality has been preliminarily examined using CDV and CPV, which needs to be validated using more virus and cell models.

## Supplementary Material

Supplementary figures and tables.

## References

[1] Montomoli E, Khadang B, Piccirella S, Trombetta C, Mennitto E, Manini I (2012). Cell culture-derived influenza vaccines from Vero cells: a new horizon for vac-cine production. Expert Rev Vaccines.

[2] Lai CC, Shih TP, Ko WC, Tang HJ, Hsueh PR (2020). Severe acute respiratory syndrome coronavirus 2 (SARS-CoV-2) and coronavirus disease-2019 (COVID-19): The epidemic and the challenges. Int J Antimicrob Agents.

[3] Wang P, Zhou R, Zhou R, Li W, Weerasinghe J, Chen S (2022). Cold atmospheric plasma for preventing infection of viruses that use ACE2 for entry. Theranostics.

[4] Cortazar OD, Megia-Macias A, Moreno S, Brun A, Gomez-Casado E (2022). Vulnerability of SARS-CoV-2 and PR8 H1N1 virus to cold atmospheric plasma activated media. Sci Rep.

[5] Hua D, Cai D, Ning M, Yu L, Zhang Z, Han P (2021). Cold atmospheric plasma selectively induces G0/G1 cell cycle arrest and apoptosis in AR-independent prostate cancer cells. J Cancer.

[6] Schneider C, Gebhardt L, Arndt S, Karrer S, Zimmermann JL, Fischer MJM (2018). Cold atmospheric plasma causes a calcium influx in melanoma cells triggering CAP-induced senescence. Sci Rep.

[7] Miao Y, Han P, Hua D, Zhou R, Guan Z, Lv Q (2021). Cold atmospheric plasma increases IBRV titer in MDBK cells by orchestrating the host cell network. Virulence.

[8] Xiang L, Xu X, Zhang S, Cai D, Dai X (2018). Cold atmospheric plasma conveys selectivity on triple negative breast cancer cells both *in vitro* and *in vivo*. Free Radic Biol Med.

[9] Furuta T, Shi L, Toyokuni S (2018). Non-thermal plasma as a simple ferroptosis inducer in cancer cells: A possible role of ferritin. Pathol Int.

[10] Bekeschus S, Eisenmann S, Sagwal SK, Bodnar Y, Moritz J, Poschkamp B (2020). xCT (SLC7A11) expression confers intrinsic resistance to physical plasma treatment in tumor cells. Redox Biol.

[11] Lin A, Gorbanev Y, De Backer J, Van Loenhout J, Van Boxem W, Lemiere F (2019). Non-Thermal Plasma as a Unique Delivery System of Short-Lived Reactive Oxygen and Nitrogen Species for Immunogenic Cell Death in Melanoma Cells. Adv Sci (Weinh).

[12] Virard F, Cousty S, Cambus JP, Valentin A, Kemoun P, Clement F (2015). Cold Atmospheric Plasma Induces a Predominantly Necrotic Cell Death via the Microenvironment. PLoS One.

[13] Dai X, Zhang Z, Zhang J, Ostrikov KK (2020). Dosing: the key to precision plasma oncology. Plasma Process Polym.

[14] Liu K, Duan Q, Zheng Z, Zhou R, Zhou R, Tang W (2021). Gas-phase peroxynitrite generation using dielectric barrier discharge at atmospheric pressure: A prospective sterilizer. Plasma Process Polym.

[15] Liu K, Hu Y, Lei J (2017). The chemical product mode transition of the air DBD driven by AC power: A plausible evaluation parameter and the chemical behaviors. Phys Plasmas.

[16] Liu K, Lei J, Zheng Z, Zhu Z, Liu S (2018). The hydrophilicity improvement of polytetrafluoroethylene by Ar plasma jet: The relationship of hydrophilicity, ambient humidity and plasma parameters. Appl Surf Sci.

[17] Liu K, Ren W, Ran CF, Zhou RS, Tang WB, Zhou RW (2021). Long-lived species in plasma-activated water generated by an AC multi-needle-to-water discharge: effects of gas flow on chemical reactions. J Physics D Appl Phys.

[18] Liu K, Xia H, Yang M, Geng W, Zuo J, Ostrikov K (2022). Insights into generation of OH radicals in plasma jets with constant power: The effects of driving voltage and frequency. Vacuum.

[19] Liu K, Zheng Z, Liu S, Hu Y (2019). Study on the Physical and Chemical Characteristics of DBD: The Effect of N2/O2 Mixture Ratio on the Product Regulation. Plasma Chem Plasma P.

[20] Dai X, Hakizimana O, Zhang X, Kaushik AC, Zhang J (2020). Orchestrated efforts on host network hijacking: Processes governing virus replication. Virulence.

[21] Dai X, Zhang X, Han P, Zhang J (2020). Canine parvovirus induces G1/S cell cycle arrest that involves EGFR Tyr1086 phosphorylation. Virulence.

[22] Mortazavi A, Williams BA, McCue K, Schaeffer L, Wold B (2008). Mapping and quantifying mammalian transcriptomes by RNA-Seq. Nat Methods.

[23] Young MD, Wakefield MJ, Smyth GK, Oshlack A (2010). Gene ontology analysis for RNA-seq: accounting for selection bias. Genome Biol.

[24] Kanehisa M, Goto S (2000). KEGG: kyoto encyclopedia of genes and genomes. Nucleic Acids Res.

[25] Yu G, Wang LG, Han Y, He QY (2012). clusterProfiler: an R package for comparing biological themes among gene clusters. OMICS.

[26] Dai X, Bazaka K, Richard DJ, Thompson ERW, Ostrikov KK (2018). The Emerging Role of Gas Plasma in Oncotherapy. Trends in biotechnology.

[27] Yang X, Chen G, Yu KN, Yang M, Peng S, Ma J (2020). Cold atmospheric plasma induces GSDME-dependent pyroptotic signaling pathway via ROS generation in tumor cells. Cell Death Dis.

[28] Kroemer G, Mariño G, Levine B (2010). Autophagy and the integrated stress response. Molecular cell.

[29] Lippai M, Lőw P (2014). The role of the selective adaptor p62 and ubiquitin-like proteins in autophagy. BioMed research international.

[30] Maruyama Y, Sou YS, Kageyama S, Takahashi T, Ueno T, Tanaka K (2014). LC3B is indispensable for selective autophagy of p62 but not basal autophagy. Biochemical and biophysical research communications.

[31] Bresciani A, Spiezia MC, Boggio R, Cariulo C, Nordheim A, Altobelli R (2018). Quantifying autophagy using novel LC3B and p62 TR-FRET assays. PLoS One.

[32] Zeng SY, Yan QJ, Yang L, Mei QH, Lu HQ (2020). Inhibition of the ROS-EGFR Pathway Mediates the Protective Action of Nox1/4 Inhibitor GKT137831 against Hypertensive Cardiac Hypertrophy via Suppressing Cardiac Inflammation and Activation of Akt and ERK1/2. Mediators Inflamm.

[33] Pudelek M, Krol K, Catapano J, Wrobel T, Czyz J, Ryszawy D (2020). Epidermal Growth Factor (EGF) Augments the Invasive Potential of Human Glioblastoma Multiforme Cells via the Activation of Collaborative EGFR/ROS-Dependent Signaling. Int J Mol Sci.

[34] Oh HN, Lee MH, Kim E, Kwak AW, Yoon G, Cho SS (2020). Licochalcone D Induces ROS-Dependent Apoptosis in Gefitinib-Sensitive or Resistant Lung Cancer Cells by Targeting EGFR and MET. Biomolecules.

[35] Chen L, Zhou Y, Tang X, Yang C, Tian Y, Xie R (2019). EGFR mutation decreases FDG uptake in nonsmall cell lung cancer via the NOX4/ROS/GLUT1 axis. Int J Oncol.

[36] Tan BL, Norhaizan ME, Chan LC (2018). ROS-Mediated Mitochondrial Pathway is Required for Manilkara Zapota (L.) P. Royen Leaf Methanol Extract Inducing Apoptosis in the Modulation of Caspase Activation and EGFR/NF-kappaB Activities of HeLa Human Cervical Cancer Cells. Evid Based Complement Alternat Med.

[37] Wang G, Li Y, Yang Z, Xu W, Yang Y, Tan X (2018). ROS mediated EGFR/MEK/ERK/HIF-1alpha Loop Regulates Glucose metabolism in pancreatic cancer. Biochemical and biophysical research communications.

[38] Lee JY, Kapur M, Li M, Choi MC, Choi S, Kim HJ (2014). MFN1 deacetylation activates adaptive mitochondrial fusion and protects metabolically challenged mitochondria. J Cell Sci.

[39] Qi Z, Shi W, Zhao Y, Ji X, Liu KJ (2019). Zinc accumulation in mitochondria promotes ischemia-induced BBB disruption through Drp1-dependent mitochondria fission. Toxicol Appl Pharmacol.

[40] Ji HW, Kim H, Kim HW, Yun SH, Park JE, Choi EH (2020). Genome-Wide Comparison of the Target Genes of the Reactive Oxygen Species and Non-Reactive Oxygen Species Constituents of Cold Atmospheric Plasma in Cancer Cells. Cancers (Basel).

[41] Dokala A, Thakur SS (2017). Extracellular region of epidermal growth factor receptor: a potential target for anti-EGFR drug discovery. Oncogene.

[42] Yim WW, Mizushima N (2020). Lysosome biology in autophagy. Cell Discov.

[43] Mathiassen SG, De Zio D, Cecconi F (2017). Autophagy and the Cell Cycle: A Complex Landscape. Front Oncol.

[44] Heo S, Kim S, Kang D (2020). The Role of Hydrogen Peroxide and Peroxiredoxins throughout the Cell Cycle. Antioxidants (Basel).

[45] Huang B, Chen Q, Wang L, Gao X, Zhu W, Mu P (2020). Aflatoxin B1 Induces Neurotoxicity through Reactive Oxygen Species Generation, DNA Damage, Apoptosis, and S-Phase Cell Cycle Arrest. Int J Mol Sci.

[46] Karuna A, Masia F, Chappell S, Errington R, Hartley AM, Jones DD (2020). Quantitative Imaging of B1 Cyclin Expression Across the Cell Cycle Using Green Fluorescent Protein Tagging and Epifluorescence. Cytometry A.

[47] Enserink JM, Kolodner RD (2010). An overview of Cdk1-controlled targets and processes. Cell Div.

[48] Montalto FI, De Amicis F (2020). Cyclin D1 in Cancer: A Molecular Connection for Cell Cycle Control, Adhesion and Invasion in Tumor and Stroma. Cells.

[49] Baker SJ, Reddy EP (2012). CDK4: A Key Player in the Cell Cycle, Development, and Cancer. Genes Cancer.

[50] Dai X, Zhang X, Ostrikov KK, Abrahamyan L (2020). Host Receptors: The Key to Establishing Cells with Broad Viral Tropism for Vaccine Production. Crit Rev Microbiol.

[51] Eucker TP, Konkel ME (2012). The cooperative action of bacterial fibronectin-binding proteins and secreted proteins promote maximal Campylobacter jejuni invasion of host cells by stimulating membrane ruffling. Cell Microbiol.

[52] Sturla LM, Amorino G, Alexander MS, Mikkelsen RB, Valerie K, Schmidt-Ullrichr RK (2005). Requirement of Tyr-992 and Tyr-1173 in phosphorylation of the epidermal growth factor receptor by ionizing radiation and modulation by SHP2. J Biol Chem.

[53] Bereiter-Hahn J, Voth M (1994). Dynamics of mitochondria in living cells: shape changes, dislocations, fusion, and fission of mitochondria. Microsc Res Tech.

[54] Liu X, Hajnoczky G (2011). Altered fusion dynamics underlie unique morphological changes in mitochondria during hypoxia-reoxygenation stress. Cell Death Differ.

[55] Youle RJ, Karbowski M (2005). Mitochondrial fission in apoptosis. Nat Rev Mol Cell Biol.

[56] Perfettini JL, Roumier T, Kroemer G (2005). Mitochondrial fusion and fission in the control of apoptosis. Trends Cell Biol.

[57] Guo H, Jiang N, Wang H, Shang K, Lu N, Li J (2019). Enhanced catalytic performance of graphene-TiO2 nanocomposites for synergetic degradation of fluoroquinolone antibiotic in pulsed discharge plasma system. Appl Catal B-Environ.

[58] Aboubakr HA, Gangal U, Youssef MM, Goyal SM, Bruggeman PJ (2016). Inactivation of virus in solution by cold atmospheric pressure plasma: identification of chemical inactivation pathways. J Phys D Appl Phys.

[59] Greenstock CL, Miller RW (1975). The oxidation of tiron by superoxide anion. Kinetics of the reaction in aqueous solution in chloroplasts. Biochim Biophys Acta.

[60] Jakovljevic A, Andric M, Miletic M, Beljic-Ivanovic K, Knezevic A, Mojsilovic S (2016). Epstein-Barr virus infection induces bone resorption in apical periodontitis via increased production of reactive oxygen species. Med Hypotheses.

[61] Ciccarone F, Di Leo L, Lazzarino G, Maulucci G, Di Giacinto F, Tavazzi B (2020). Aconitase 2 inhibits the proliferation of MCF-7 cells promoting mitochondrial oxidative metabolism and ROS/FoxO1-mediated autophagic response. Br J Cancer.

[62] Gao Y, Yang Y, Yuan F, Huang J, Xu W, Mao B (2017). TNFalpha-YAP/p65-HK2 axis mediates breast cancer cell migration. Oncogenesis.

