# Supplementary material for: Cold Atmospheric Plasma Boosts Virus Multiplication via EGFR(Tyr1068) Phosphorylation-Mediated Control on Cell Mitophagy
Source: Int J Biol Sci. 2022 May 9;18(8):3405–20. doi: 10.7150/ijbs.71983 (PMC9134911; doi:10.7150/ijbs.71983)
Supplement: Supplementary file 1 — Supplementary figures and tables. [file ijbsv18p3405s1.pdf]

Supplementary Figures

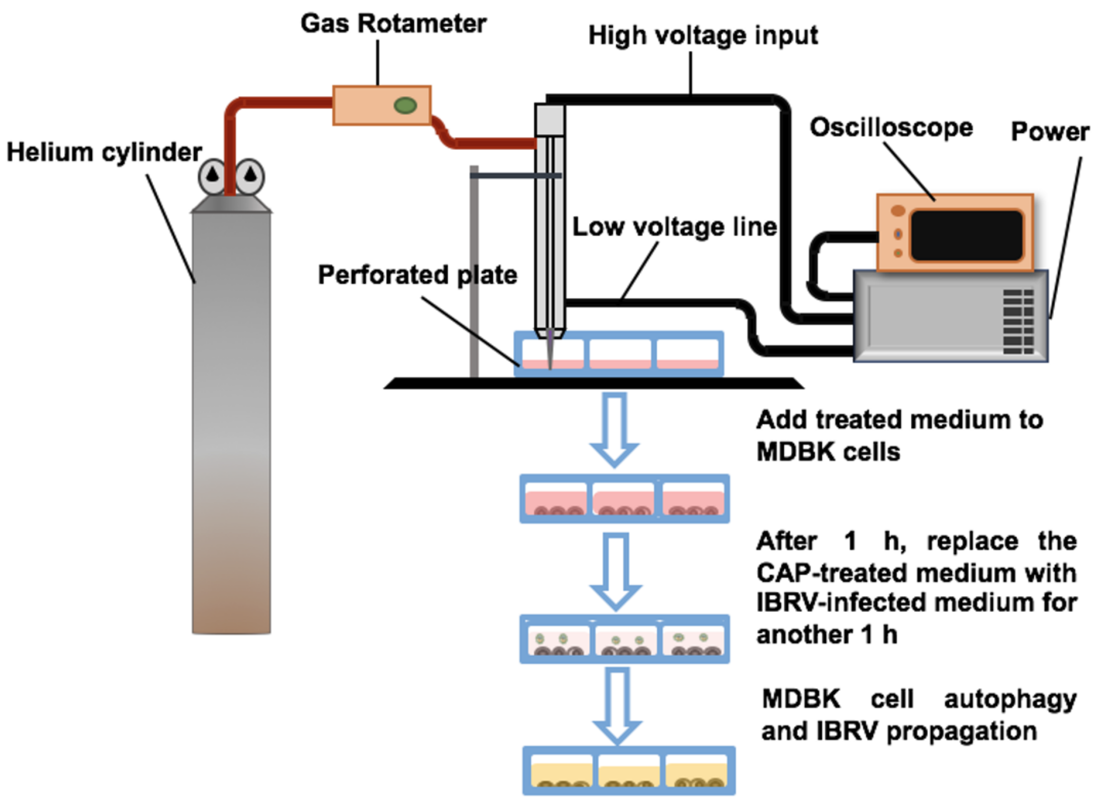

Supplementary Figure 1. Illustration on CAP device and experimental design.

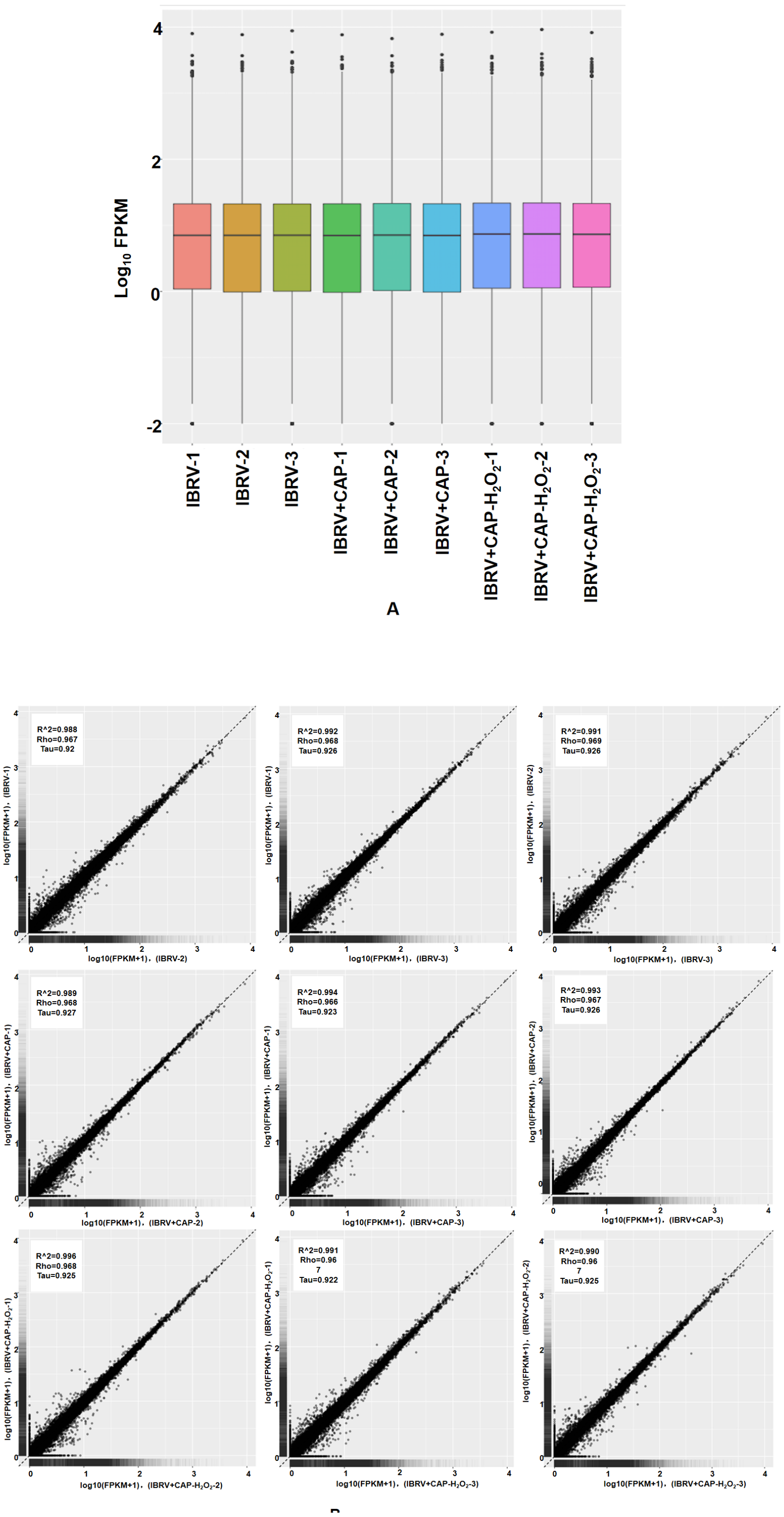

Supplementary Figure 2. Quality control results of the transcriptomics data. **(A)** FPKM plot, and **(B)** pair-wise Pearson correlations for samples sent for transcriptome sequencing.

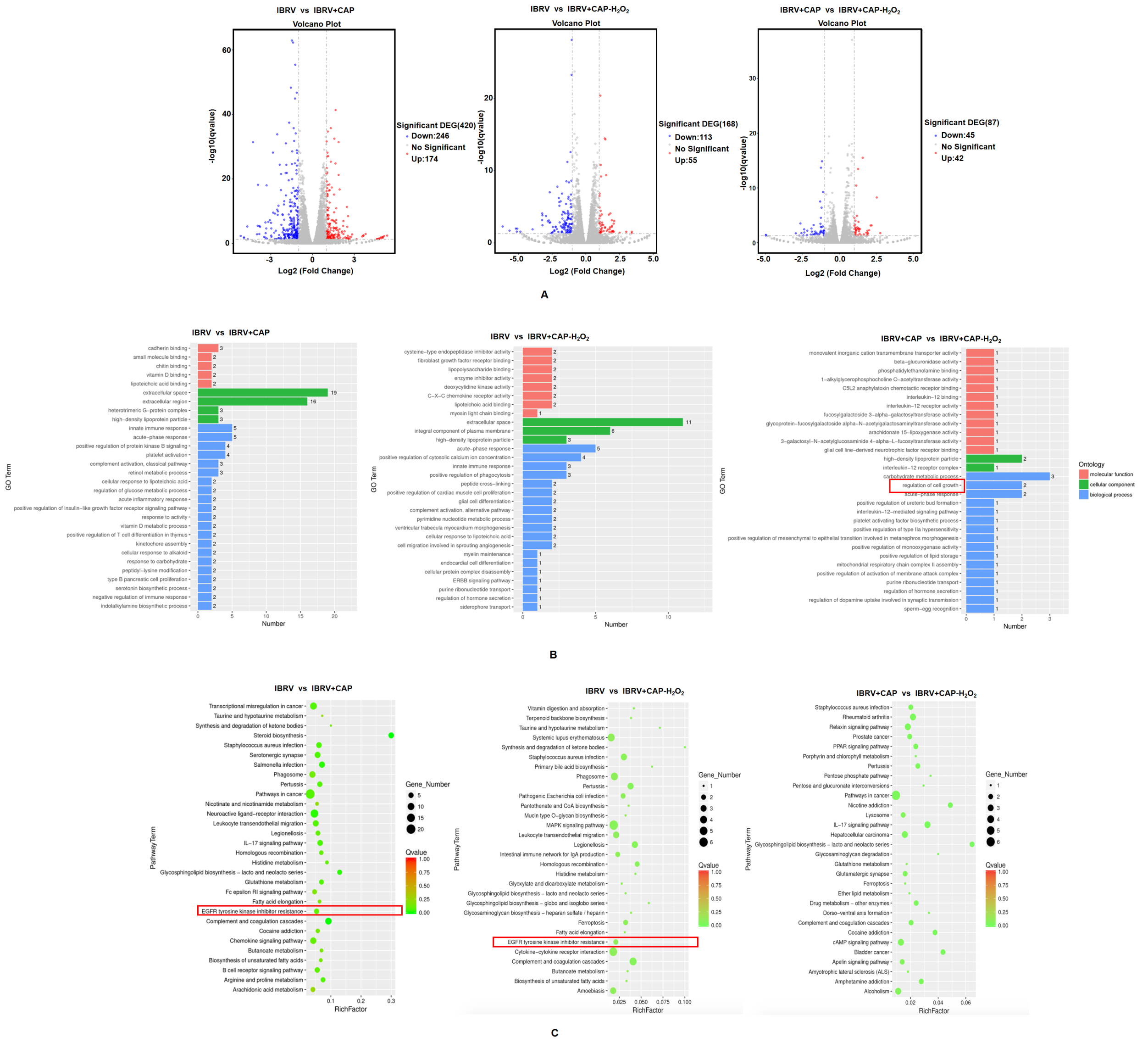

Supplementary Figure 3. Identification of differentially expressed genes, Gene ontology enrichment analysis, KEGG pathway analysis across sample groups. **(A)** Volcano plots showing genes differentially expressed between sample groups. **(B)** GO terms and **(C)** KEGG pathways enriched with genes differentially expressed between sample groups. There are 3 sample groups: IBRV, IBRV+CAP, IBRV+CAP-H_2O_2. IBRV: IBRV-infected MDBK cells infected with IBRV, IBRV+CAP: IBRV-infected MDBK cells treated with CAP; IBRV+CAP-H_2O_2: IBRV-infected MDBK cells treated with CAP and H_2O_2 scavenger.

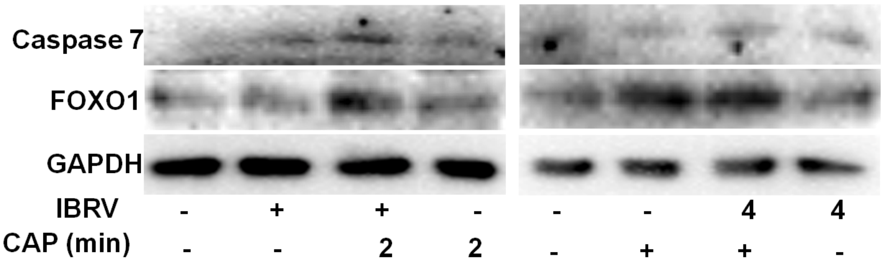

A

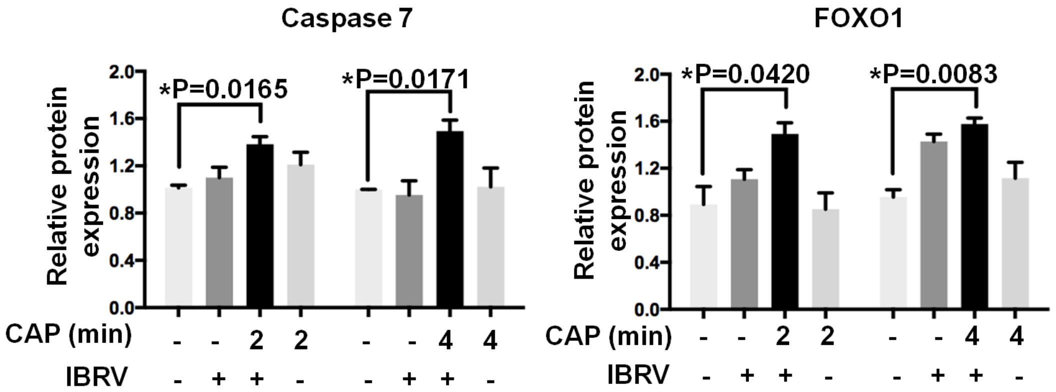

Supplementary Figure 4. Protein expression of representative proteins in cell apoptosis and anti-oxidative ability control on CAP exposure, IBRV infection and joint treatment. **(A)** Western blot. **(B)** Quantification. Caspase 7 and FOXO1 are representative proteins of cell apoptosis and anti-oxidative ability.

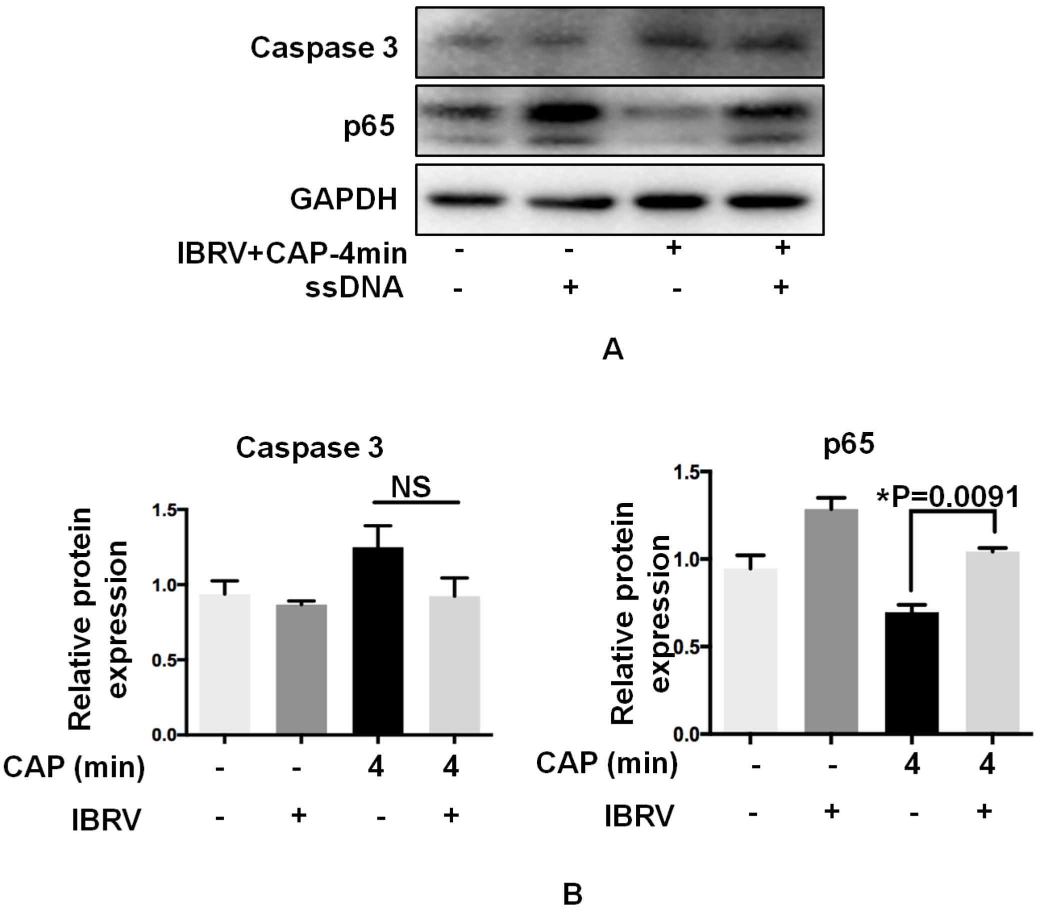

Supplementary Figure 5. Efficacy of ssDNA targeting EGFR(Tyr1068) in inducing relapsed expression of representative proteins involved in cell apoptosis and migration. **(A)** Western blot. **(B)** Quantification. Caspase 3 and p65 are representative proteins of cell apoptosis and migration.

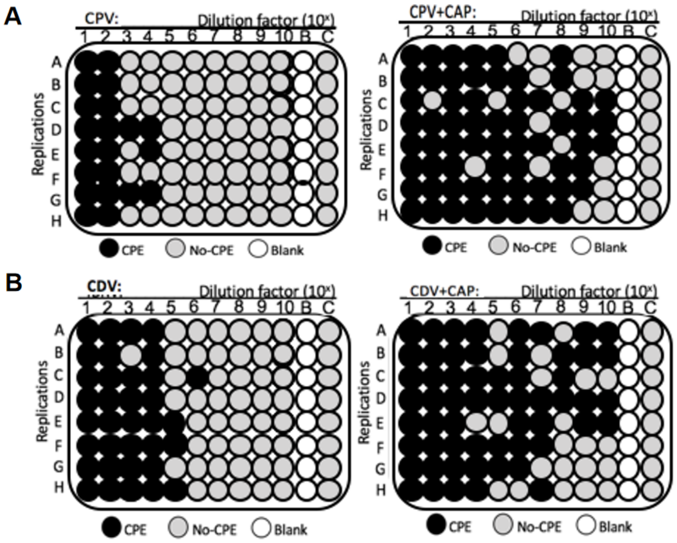

Titer (TCID50/0.1mL) of CPV treated by plasma and H_2O_2 scavenger

Groups	TCID50/0.1mL
CPV	$10^{-3.125}$
CPV+CAP	$10^{-8.25}$

Titer (TCID50/0.1mL) of CPV treated by plasma and H_2O_2 scavenger

Groups	TCID50/0.1mL
CDV	$10^{-4.875}$
CDV+CAP	$10^{-7.75}$

Supplementary Figure 6. TCID50 results showing the effect of CAP on CPV and CDV virus titer after infecting MDCK cells. **(A)** CPV. **(B)** CDV.

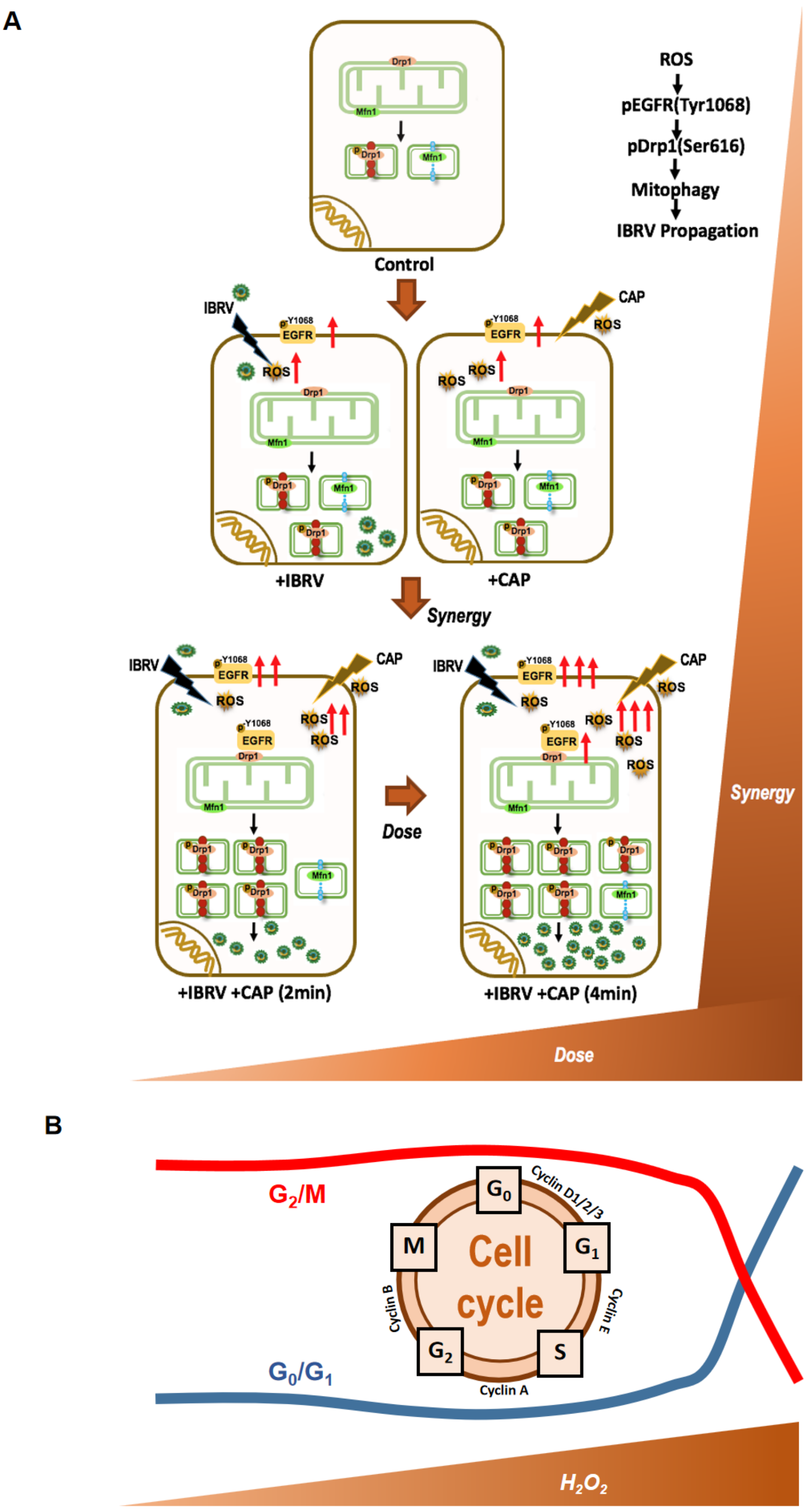
A
ROS
pEGFR(Tyr1068)
pDrp1(Ser616)
Mitophagy
IBRV Propagation
Control
Drp1
Mfn1
IBRV
CAP
EGFR
Y1068
ROS
+IBRV
+CAP
Synergy
Dose
+IBRV +CAP (2min)
+IBRV +CAP (4min)
Synergy
Dose
B
G_2/M
G_0/G_1
Cell cycle
G_0
G_1
S
G_2
M
Cyclin D1/2/3
Cyclin E
Cyclin A
Cyclin B
H_2O_2

Supplementary Figure 7. Conceptual illustration on the role of CAP in triggering mitophagy via EGFR (Tyr1068) phosphorylation induced mitochondria fission. **(A)** Both IBRV infection and CAP exposure could enhance cellular ROS level, and their synergistic effect could substantially increase EGFR(Tyr1068) phosphorylation that is translocated into the surrounding region of mitochondria and interacts with Drp1(Ser616). Enhanced level of phosphorylated EGFR(Tyr1086) is associated with elevated Drp1(Ser616), the physical interaction of which leads to boosted mitochondria fission. Halted cell growth as a result of mitophagy fosters a favorable environment for virus propagation, and the effect increases with CAP treatment duration before cellular ROS level reaches the apoptotic threshold. **(B)** Cells first undergo slightly decreased and later dramatically increased G_0/G_1 cell cycle arrest, and slightly enhanced and later substantially reduced G_2/M cell cycle stage with the increase of H_2O_2 concentration.

Supplementary Tables

Supplementary Table 1. Primers for IBRV detection and EGFR mRNA quantification.

Gene name		Sequence (5’-3’)
IBRV	Forward Primer	CGTGGTGGTGCCAGTTAG
	Reverse Primer	TCATCGTCGCTGTCGTCAT
EGFR	Forward Primer	GGGAGCTACCCTCTCAAGGA
	Reverse Primer	GGGGCTGGAAGTTTATGGCT

Supplementary Table 2. Details of protein antibodies used in this study.

Type	Protein	Experiment	Supplier name	Catalog number	Clone name
Primary antibody	LC3B	WB, IF	Abcam	ab48394	Rabbit
	P62	WB	Cell Signaling Technology	#39749	Rabbit
	EGFR	WB	Cell Signaling Technology	#4267	Rabbit
	p-EGFR(Y1068)	WB, IF	Cell Signaling Technology	#3777	Rabbit
	p-EGFR(Y1086)	WB	Abcam	ab32086	Rabbit
	p-EGFR(Y845)	WB	Cell Signaling Technology	#2231	Rabbit
	GAPDH	WB	Proteitech	AC001	Rabbit
	p-Drp 1 (Ser616)	WB, IF	Cell Signaling Technology	#3455	Rabbit
	Mfn 1	WB	Santa Cruz	sc-166644	Mouse
	FOXO1	WB	Cell Signaling Technology	#2880	Rabbit
	P65	WB	Cell Signaling Technology	#8242	Rabbit
	Caspase 7	WB	Abcam	ab256469	Rabbit
	Caspase 3	WB	Abcam	Ab32351	Rabbit
Secondary antibody	HRP goat anti-rabbit IgG (H+L)	WB	Beyotime	A0208	Goat
	HRP goat anti-mouse IgG (H+L)	WB	Beyotime	A0216	Goat
	Immunol Fluorescence staining kit, anti-rabbit Alexa Fluor 488	IF	Beyotime	P0176-1	Goat

Supplementary Table 3. List of differentially expressed genes in different pair-wise group comparisons. There are 3 sample groups: IBRV, IBRV+CAP, IBRV+CAP-H_2O_2. IBRV: IBRV-infected MDBK cells infected with IBRV, IBRV+CAP: IBRV-infected MDBK cells treated with CAP; IBRV+CAP-H_2O_2: IBRV-infected MDBK cells treated with CAP and H_2O_2 scavenger.

Enzembl ID	Gene Symbol	IBRV vs IBRV+CAP			IBRV vs IBRV+CAP-H2O2			IBRV+CAP vs IBRV+CAP-H2[illegible]		
		p value	FDR	Regulation	p value	FDR	Regulation	p value	FDR	Regulatio
ENSBTAG00000000020	TRPV3	0.0063	0.0492	Down						
ENSBTAG00000000062	RASL12	0.0001	0.0019	Down	0.0001	0.0025	Down			
ENSBTAG00000000082	KCNJ14				0.0000	0.0001	Down			
ENSBTAG00000000123	HAO2	0.0000	0.0000	Down	0.0001	0.0049	Down			
ENSBTAG00000000344	SPACA3							0.0005	0.0211	Up
ENSBTAG00000000414	FUT6	0.0000	0.0000	Down				0.0000	0.0025	Up
ENSBTAG00000000437	FFAR4	0.0000	0.0000	Down						
ENSBTAG00000000507	NR4A1				0.0000	0.0015	Down	0.0000	0.0000	Down
ENSBTAG00000000522	AHSG	0.0000	0.0000	Down	0.0000	0.0001	Down			
ENSBTAG00000000570	RET	0.0038	0.0327	Down						
ENSBTAG00000000580	LY6G6F	0.0000	0.0000	Up	0.0000	0.0007	Up			
ENSBTAG00000000604	GPNMB	0.0000	0.0000	Down	0.0000	0.0000	Down			
ENSBTAG00000000629	MMS22L	0.0000	0.0000	Up						
ENSBTAG00000000654	ARMC4							0.0000	0.0013	Down
ENSBTAG00000000706	ADAMT1	0.0000	0.0001	Down	0.0000	0.0000	Down			
ENSBTAG00000000835	BCAS1	0.0000	0.0000	Down	0.0000	0.0004	Down			
ENSBTAG00000000959	ZSWIM1	0.0000	0.0000	Down						

ENSBTAG00000000961	NEURL2	0.0001	0.0009	Down						
ENSBTAG00000000987	OTOG	0.0043	0.0365	Up						
ENSBTAG00000001060	CXCR4	0.0000	0.0000	Up	0.0000	0.0000	Up			
ENSBTAG00000001305	ATP2B2	0.0000	0.0000	Down						
ENSBTAG00000001538	-				0.0000	0.0003	Down	0.0000	0.0004	Down
ENSBTAG00000001592	INSIG1	0.0000	0.0000	Down	0.0000	0.0000	Down			
ENSBTAG00000001765	HAPLN4				0.0000	0.0000	Down			
ENSBTAG00000001785	TGM3	0.0000	0.0000	Down	0.0000	0.0004	Down			
ENSBTAG00000001804	GPR179	0.0057	0.0457	Down						
ENSBTAG00000001920	POLQ				0.0000	0.0000	Up			
ENSBTAG00000002144	ADRB2	0.0005	0.0060	Up						
ENSBTAG00000002163	PTGER2	0.0049	0.0406	Down						
ENSBTAG00000002340	STEAP4	0.0000	0.0001	Down						
ENSBTAG00000002464	PLCH2	0.0002	0.0029	Up						
ENSBTAG00000002478	AGMAT	0.0000	0.0000	Up						
ENSBTAG00000002497	ELOVL2	0.0000	0.0000	Down	0.0000	0.0000	Down			
ENSBTAG00000002555	PDZK1I1	0.0000	0.0000	Down						
ENSBTAG00000002765	CYP24A1	0.0000	0.0000	Down						
ENSBTAG00000002914	GALNT18	0.0003	0.0036	Down	0.0000	0.0009	Down			
ENSBTAG00000002919	UNC5CL	0.0000	0.0000	Down						
ENSBTAG00000002965	NEURL3	0.0000	0.0000	Down						
ENSBTAG00000003169	FBXO24	0.0000	0.0000	Down						
ENSBTAG00000003192	TBC1D16	0.0001	0.0011	Up						
ENSBTAG00000003212	NNAT	0.0002	0.0033	Down						
ENSBTAG00000003467	SLC5A11	0.0038	0.0332	Down	0.0017	0.0314	Down			

ENSBTAG00000003474	RAB15	0.0000	0.0000	Down						
ENSBTAG00000003700	SLC5A10	0.0002	0.0028	Down						
ENSBTAG00000003707	GRAP	0.0000	0.0004	Down				0.0001	0.0058	Up
ENSBTAG00000003711	EPAS1	0.0000	0.0000	Down						
ENSBTAG00000003871	CYP2B6	0.0000	0.0000	Up	0.0000	0.0012	Up			
ENSBTAG00000003956	SGCA	0.0062	0.0489	Down						
ENSBTAG00000003977	SLC52A3	0.0000	0.0000	Down	0.0000	0.0000	Down			
ENSBTAG00000004010	PAPPA	0.0000	0.0000	Down	0.0000	0.0004	Down			
ENSBTAG00000004014	FBLN2	0.0004	0.0054	Up						
ENSBTAG00000004099	CCDC170	0.0056	0.0451	Up						
ENSBTAG00000004150	NRG1	0.0000	0.0000	Up						
ENSBTAG00000004221	ESM1	0.0000	0.0000	Up	0.0001	0.0030	Up			
ENSBTAG00000004246	H6PD	0.0000	0.0000	Down				0.0001	0.0047	Up
ENSBTAG00000004362	SERPIN1	0.0000	0.0005	Down						
ENSBTAG00000004572	RNF183	0.0000	0.0000	Down						
ENSBTAG00000004657	FBLL1	0.0000	0.0003	Down	0.0001	0.0022	Down			
ENSBTAG00000004688	DHCR24	0.0000	0.0000	Down						
ENSBTAG00000004761	FOXH1				0.0029	0.0464	Up			
ENSBTAG00000004840	C1S	0.0000	0.0000	Down						
ENSBTAG00000005092	ROR2	0.0000	0.0000	Down	0.0002	0.0062	Down			
ENSBTAG00000005122	KNG1	0.0000	0.0000	Down	0.0001	0.0029	Down			
ENSBTAG00000005164	GZMK	0.0000	0.0002	Up	0.0002	0.0062	Up			
ENSBTAG00000005176	GDNF							0.0016	0.0464	Up
ENSBTAG00000005251	-				0.0019	0.0349	Down			
ENSBTAG00000005390	GMFG	0.0005	0.0056	Up				0.0009	0.0313	Down

ENSBTAG00000005456	TTK	0.0000	0.0000	Up						
ENSBTAG00000005477	LAPTM5							0.0000	0.0016	Up
ENSBTAG00000005498	SQLE	0.0000	0.0000	Down						
ENSBTAG00000005691	FGF2	0.0001	0.0011	Up	0.0013	0.0260	Up			
ENSBTAG00000005828	MERTK				0.0000	0.0001	Down			
ENSBTAG00000005936	DMC1	0.0000	0.0004	Up	0.0014	0.0280	Up			
ENSBTAG00000006135	MAP1LC 3A							0.0003	0.0153	Up
ENSBTAG00000006152	-	0.0017	0.0175	Up	0.0001	0.0033	Up			
ENSBTAG00000006221	ADGRG3	0.0009	0.0103	Down	0.0000	0.0016	Down			
ENSBTAG00000006367	CCN2							0.0000	0.0000	Down
ENSBTAG00000006377	MYO1G	0.0000	0.0000	Up						
ENSBTAG00000006387	TSNAXI1	0.0001	0.0014	Down						
ENSBTAG00000006404	CENPT	0.0000	0.0000	Up						
ENSBTAG00000006447	ACSM3	0.0038	0.0328	Up						
ENSBTAG00000006523	SOD2	0.0000	0.0000	Down						
ENSBTAG00000006686	NPNT	0.0000	0.0000	Down						
ENSBTAG00000006961	NLRP13	0.0001	0.0018	Up				0.0002	0.0096	Down
ENSBTAG00000007024	-	0.0024	0.0226	Down						
ENSBTAG00000007129	MRVI1	0.0019	0.0188	Up						
ENSBTAG00000007241	SLAMF9	0.0000	0.0003	Up						
ENSBTAG00000007388	ZC3H12D	0.0000	0.0000	Down	0.0000	0.0021	Down			
ENSBTAG00000007449	HS3ST1	0.0000	0.0000	Down	0.0000	0.0001	Down			
ENSBTAG00000007450	C2	0.0004	0.0050	Down						
ENSBTAG00000007496	P2RX2	0.0055	0.0445	Down						

ENSBTAG00000007589	SMAD9	0.0000	0.0001	Down						
ENSBTAG00000007638	PIF1	0.0000	0.0000	Up						
ENSBTAG00000007732	ARPP21	0.0000	0.0000	Up						
ENSBTAG00000007879	SRRM3	0.0016	0.0161	Up						
ENSBTAG00000007958	TNS4	0.0000	0.0000	Up	0.0000	0.0000	Up			
ENSBTAG00000008111	ESYT3	0.0014	0.0146	Up				0.0003	0.0138	Down
ENSBTAG00000008182	FOSB	0.0000	0.0000	Up						
ENSBTAG00000008333	ETV4	0.0000	0.0000	Up						
ENSBTAG00000008538	DNAI1	0.0029	0.0266	Up						
ENSBTAG00000008546	SYNGR4	0.0000	0.0000	Up	0.0012	0.0251	Up			
ENSBTAG00000008587	GSTT2	0.0001	0.0019	Down				0.0016	0.0461	Up
ENSBTAG00000008611	IGFBP4	0.0000	0.0000	Up	0.0000	0.0000	Up			
ENSBTAG00000008612	C1R	0.0000	0.0000	Down	0.0000	0.0001	Down	0.0000	0.0008	Up
ENSBTAG00000008652	DUSP27	0.0000	0.0000	Up						
ENSBTAG00000008739	SAMD11	0.0000	0.0000	Down						
ENSBTAG00000008779	ALOX1B	0.0000	0.0001	Down				0.0001	0.0081	Up
ENSBTAG00000008788	SLC13A2	0.0000	0.0000	Down						
ENSBTAG00000008827	SPOCK2	0.0000	0.0001	Down				0.0010	0.0323	Up
ENSBTAG00000009115	SPSB4				0.0006	0.0148	Down			
ENSBTAG00000009124	FEZ1	0.0000	0.0000	Up						
ENSBTAG00000009455	IL12RB2	0.0000	0.0000	Down				0.0004	0.0163	Up
ENSBTAG00000009513	TGFBI	0.0021	0.0205	Up	0.0003	0.0083	Up			
ENSBTAG00000009673	SLC9B1	0.0035	0.0307	Up						
ENSBTAG00000009867	FAM71F1	0.0000	0.0000	Up	0.0002	0.0067	Up	0.0001	0.0076	Down
ENSBTAG00000010062	SLC26A4	0.0000	0.0003	Down						

ENSBTAG00000010116	AK9	0.0011	0.0119	Up						
ENSBTAG00000010238	LTB4R	0.0000	0.0001	Up						
ENSBTAG00000010245	SPRY3	0.0000	0.0000	Down	0.0000	0.0000	Down			
ENSBTAG00000010422	MDM2	0.0000	0.0000	Up						
ENSBTAG00000010517	EVPL	0.0000	0.0000	Down				0.0003	0.0142	Up
ENSBTAG00000010564	ELOVL6	0.0000	0.0000	Down						
ENSBTAG00000010622	TRIM46	0.0000	0.0000	Down						
ENSBTAG00000010726	F8	0.0000	0.0002	Down						
ENSBTAG00000010896	NKX3-2	0.0063	0.0497	Up				0.0001	0.0067	Down
ENSBTAG00000010991	TTR	0.0002	0.0033	Down						
ENSBTAG00000011237	PKHD1	0.0000	0.0000	Down						
ENSBTAG00000011381	SLC30A3				0.0000	0.0002	Down			
ENSBTAG00000011398	PRR7				0.0021	0.0365	Down			
ENSBTAG00000011409	CD72	0.0043	0.0365	Down	0.0029	0.0473	Down			
ENSBTAG00000011511	-	0.0000	0.0000	Up						
ENSBTAG00000011636	DDIAS	0.0000	0.0000	Up	0.0000	0.0000	Up			
ENSBTAG00000011640	PLPP3	0.0000	0.0000	Down						
ENSBTAG00000011648	TMLHE	0.0000	0.0000	Down						
ENSBTAG00000011693	LENG9	0.0001	0.0012	Down						
ENSBTAG00000011839	HMGCS1	0.0000	0.0000	Down	0.0000	0.0000	Down			
ENSBTAG00000011927	RDH5	0.0000	0.0003	Down						
ENSBTAG00000011938	SLC25A2	0.0001	0.0010	Down				0.0001	0.0054	Up
ENSBTAG00000011971	NRP2	0.0000	0.0000	Up						
ENSBTAG00000012032	PDE4A							0.0018	0.0496	Down
ENSBTAG00000012052	PADI4							0.0000	0.0002	Up

ENSBTAG00000012066	PECAM1	0.0001	0.0016	Up						
ENSBTAG00000012150	-	0.0059	0.0471	Up						
ENSBTAG00000012164	CP	0.0000	0.0000	Down	0.0000	0.0006	Down			
ENSBTAG00000012249	PRR15L	0.0000	0.0000	Down						
ENSBTAG00000012351	ARNT2	0.0000	0.0000	Down						
ENSBTAG00000012432	FDFT1	0.0000	0.0000	Down						
ENSBTAG00000012443	DIAPH3	0.0000	0.0000	Up						
ENSBTAG00000012525	ABO	0.0000	0.0000	Down				0.0000	0.0001	Up
ENSBTAG00000012979	ZMYND2	0.0008	0.0086	Up	0.0001	0.0043	Up			
ENSBTAG00000013066	IGF2	0.0000	0.0000	Down						
ENSBTAG00000013250	PCSK1N	0.0025	0.0235	Up						
ENSBTAG00000013320	TSPAN1	0.0000	0.0000	Down						
ENSBTAG00000013338	SLC7A9	0.0008	0.0086	Up	0.0005	0.0125	Up			
ENSBTAG00000013364	LIPM				0.0001	0.0043	Down			
ENSBTAG00000013496	CPM	0.0000	0.0000	Down						
ENSBTAG00000013511	VWA5B1	0.0001	0.0014	Down	0.0029	0.0464	Down			
ENSBTAG00000013578	CHI3L2	0.0000	0.0001	Down				0.0006	0.0235	Up
ENSBTAG00000013619	SCTR	0.0000	0.0004	Up				0.0000	0.0003	Down
ENSBTAG00000013670	SLC46A2	0.0001	0.0017	Down						
ENSBTAG00000013718	GC	0.0009	0.0098	Down						
ENSBTAG00000013736	PROM1	0.0000	0.0001	Up						
ENSBTAG00000013798	PTPRN	0.0041	0.0353	Down						
ENSBTAG00000013836	-				0.0010	0.0216	Up			
ENSBTAG00000013861	SLC8A1	0.0045	0.0375	Up				0.0002	0.0094	Down
ENSBTAG00000013984	KL				0.0001	0.0023	Up			

ENSBTAG00000013989	JHY	0.0000	0.0000	Up	0.0000	0.0001	Up			
ENSBTAG00000014132	SNED1	0.0000	0.0000	Down						
ENSBTAG00000014149	LCN2	0.0023	0.0216	Down	0.0002	0.0071	Down			
ENSBTAG00000014189	RGS6	0.0007	0.0078	Down				0.0000	0.0031	Up
ENSBTAG00000014246	CENPH	0.0000	0.0000	Up	0.0000	0.0000	Up			
ENSBTAG00000014315	PBLD	0.0000	0.0000	Down	0.0000	0.0000	Down	0.0000	0.0000	Up
ENSBTAG00000014459	MAP6D1	0.0003	0.0034	Down						
ENSBTAG00000014486	ATP12A	0.0000	0.0000	Down				0.0000	0.0000	Up
ENSBTAG00000014496	CCR6	0.0000	0.0000	Down	0.0003	0.0095	Down			
ENSBTAG00000014508	FBXO40	0.0000	0.0000	Down	0.0000	0.0015	Down			
ENSBTAG00000014514	SEC1	0.0000	0.0000	Down	0.0007	0.0156	Down			
ENSBTAG00000014655	MYO1A	0.0006	0.0073	Up						
ENSBTAG00000014773	HMMR	0.0000	0.0000	Up						
ENSBTAG00000014821	SLC7A7	0.0000	0.0000	Down						
ENSBTAG00000014885	MYOM3	0.0000	0.0000	Down						
ENSBTAG00000015032	CD14	0.0000	0.0000	Down	0.0000	0.0001	Down			
ENSBTAG00000015094	VNN1	0.0000	0.0001	Down	0.0003	0.0074	Down			
ENSBTAG00000015208	CARMIL3	0.0000	0.0000	Up						
ENSBTAG00000015222	RSRP1	0.0000	0.0000	Down						
ENSBTAG00000015345	BNIPL	0.0045	0.0380	Down						
ENSBTAG00000015349	C3H1orf6	0.0014	0.0144	Down	0.0014	0.0279	Down			
ENSBTAG00000015351	STYK1	0.0002	0.0022	Up						
ENSBTAG00000015402	GREB1	0.0000	0.0001	Down	0.0003	0.0084	Down			
ENSBTAG00000015412	TGM7	0.0030	0.0270	Up						
ENSBTAG00000015549	PCDH18	0.0000	0.0000	Up	0.0000	0.0000	Up			

ENSBTAG00000015666	-	0.0010	0.0112	Up						
ENSBTAG00000015685	KIF12	0.0000	0.0000	Down						
ENSBTAG00000015690	PLIN4	0.0000	0.0000	Down						
ENSBTAG00000015692	HSPA4L	0.0000	0.0000	Up						
ENSBTAG00000015743	GMPR	0.0020	0.0196	Up				0.0000	0.0026	Down
ENSBTAG00000015818	-	0.0000	0.0000	Up				0.0000	0.0000	Down
ENSBTAG00000015912	DMKN	0.0000	0.0001	Up						
ENSBTAG00000015924	CALB1	0.0000	0.0000	Down						
ENSBTAG00000015981	ETV1	0.0012	0.0126	Up						
ENSBTAG00000016124	PACSIN1	0.0000	0.0000	Up						
ENSBTAG00000016159	NPBWR1	0.0013	0.0132	Down	0.0013	0.0270	Down			
ENSBTAG00000016217	RBM43							0.0000	0.0000	Up
ENSBTAG00000016276	HAL	0.0015	0.0150	Down	0.0004	0.0103	Down			
ENSBTAG00000016343	KCNIP2	0.0021	0.0199	Down						
ENSBTAG00000016399	AKAP4	0.0049	0.0407	Up						
ENSBTAG00000016465	DHCR7	0.0000	0.0000	Down						
ENSBTAG00000016523	DNAH10	0.0000	0.0000	Up						
ENSBTAG00000016805	SGMS2				0.0001	0.0024	Down			
ENSBTAG00000016819	FABP3				0.0000	0.0006	Down			
ENSBTAG00000016821	SNX22	0.0001	0.0011	Down	0.0003	0.0076	Down			
ENSBTAG00000016864	LBP	0.0000	0.0000	Down	0.0000	0.0018	Down			
ENSBTAG00000017024	PPARGCIA				0.0001	0.0027	Down			
ENSBTAG00000017026	DEPDC1 B	0.0000	0.0000	Up						
ENSBTAG00000017041	PTGER1	0.0001	0.0016	Down	0.0017	0.0319	Down			

ENSBTAG00000017104	MUC1	0.0000	0.0000	Down						
ENSBTAG00000017133	GINS4	0.0000	0.0000	Up						
ENSBTAG00000017216	FRMD5	0.0054	0.0434	Up						
ENSBTAG00000017271	MASTL	0.0000	0.0000	Up						
ENSBTAG00000017280	C3	0.0000	0.0000	Down	0.0000	0.0000	Down	0.0000	0.0000	Up
ENSBTAG00000017405	RORC	0.0001	0.0015	Up						
ENSBTAG00000017599	NR2F1				0.0000	0.0004	Down			
ENSBTAG00000017617	BNIP5	0.0036	0.0315	Down	0.0011	0.0229	Down			
ENSBTAG00000017690	CARNS1	0.0000	0.0000	Down						
ENSBTAG00000017709	ATRNL1	0.0000	0.0002	Up						
ENSBTAG00000017769	RIBC1	0.0000	0.0004	Up						
ENSBTAG00000017793	KLRG2	0.0001	0.0011	Down						
ENSBTAG00000017911	HMX2							0.0002	0.0093	Down
ENSBTAG00000018016	NUPR1	0.0000	0.0000	Down						
ENSBTAG00000018119	AOAH	0.0000	0.0000	Up						
ENSBTAG00000018133	SEMA3A	0.0000	0.0000	Up	0.0007	0.0156	Up			
ENSBTAG00000018142	DTL	0.0000	0.0000	Up						
ENSBTAG00000018167	KLHL31	0.0000	0.0000	Down						
ENSBTAG00000018172	OGFOD3	0.0000	0.0000	Down						
ENSBTAG00000018223	CHI3L1	0.0000	0.0000	Down						
ENSBTAG00000018399	MYH15				0.0000	0.0000	Up			
ENSBTAG00000018424	ACKR3				0.0000	0.0001	Down	0.0000	0.0000	Down
ENSBTAG00000018497	CAVIN2	0.0002	0.0024	Up				0.0000	0.0011	Down
ENSBTAG00000018543	VIL1	0.0016	0.0166	Down						
ENSBTAG00000018650	HEPACAM	0.0005	0.0061	Down						

ENSBTAG00000018785	TUBB1				0.0001	0.0046	Down			
ENSBTAG00000018864	CENPU	0.0000	0.0000	Up						
ENSBTAG00000018936	LSS	0.0000	0.0000	Down						
ENSBTAG00000019028	STOX1	0.0000	0.0004	Up						
ENSBTAG00000019122	EFCAB5	0.0000	0.0000	Up						
ENSBTAG00000019272	LPCAT2							0.0004	0.0160	Down
ENSBTAG00000019277	KCNH3				0.0014	0.0270	Down	0.0002	0.0117	Down
ENSBTAG00000019350	SLC25A21	0.0003	0.0040	Down						
ENSBTAG00000019378	KIAA1755				0.0005	0.0121	Down			
ENSBTAG00000019496	CDKN3	0.0000	0.0000	Up						
ENSBTAG00000019513	C11H9orf 50	0.0000	0.0000	Up						
ENSBTAG00000019628	EDAR	0.0001	0.0017	Down						
ENSBTAG00000019796	FCMR	0.0004	0.0053	Down						
ENSBTAG00000019798	PIGR	0.0000	0.0000	Down	0.0000	0.0000	Down			
ENSBTAG00000019912	CNTD1	0.0028	0.0256	Up						
ENSBTAG00000019940	RASGRF1				0.0000	0.0000	Down			
ENSBTAG00000019975	IL7R	0.0005	0.0066	Up						
ENSBTAG00000020003	KLHL14	0.0000	0.0000	Down						
ENSBTAG00000020017	SLC25A41	0.0000	0.0001	Down						
ENSBTAG00000020028	RBP1	0.0018	0.0176	Down						
ENSBTAG00000020173	INPP5D	0.0053	0.0433	Up						
ENSBTAG00000020227	FAM72A	0.0000	0.0000	Up						
ENSBTAG00000020353	PFN3	0.0060	0.0475	Up						
ENSBTAG00000020485	ARRB1	0.0000	0.0000	Down						

ENSBTAG00000020512	GJB1	0.0000	0.0000	Down	0.0000	0.0019	Down	0.0000	0.0025	Up
ENSBTAG00000020580	TCN1	0.0001	0.0021	Down						
ENSBTAG00000020589	ENTPD1	0.0020	0.0194	Up						
ENSBTAG00000020774	-	0.0001	0.0012	Down						
ENSBTAG00000020792	TPH2	0.0000	0.0000	Up						
ENSBTAG00000020839	MEGF6	0.0000	0.0000	Up						
ENSBTAG00000020869	DDC	0.0000	0.0000	Down						
ENSBTAG00000020984	RAPGEF4	0.0000	0.0000	Up	0.0021	0.0364	Up			
ENSBTAG00000020990	P2RY14	0.0000	0.0000	Down						
ENSBTAG00000021069	PBK	0.0000	0.0000	Up						
ENSBTAG00000021082	TMEM125	0.0000	0.0000	Down						
ENSBTAG00000021097	PELI3				0.0000	0.0014	Down			
ENSBTAG00000021177	ADAMTS14	0.0019	0.0190	Down	0.0017	0.0315	Down			
ENSBTAG00000021322	TM4SF20				0.0015	0.0296	Up			
ENSBTAG00000021466	COL3A1	0.0000	0.0000	Down	0.0000	0.0000	Down			
ENSBTAG00000021516	GSTA1	0.0000	0.0000	Down						
ENSBTAG00000021649	-	0.0000	0.0003	Up						
ENSBTAG00000021658	GRASP				0.0026	0.0432	Down	0.0006	0.0226	Down
ENSBTAG00000021681	PRR11	0.0000	0.0000	Up						
ENSBTAG00000021842	FCGR2B	0.0000	0.0000	Down						
ENSBTAG00000021924	PLA2G4E	0.0014	0.0144	Up						
ENSBTAG00000021945	NID2	0.0000	0.0000	Up						
ENSBTAG00000021995	LRTM2				0.0002	0.0053	Down			

ENSBTAG00000022120	FGB	0.0001	0.0011	Down						
ENSBTAG00000022394	SAA2	0.0000	0.0000	Down	0.0001	0.0038	Down			
ENSBTAG00000022395	-	0.0000	0.0000	Down	0.0002	0.0058	Down			
ENSBTAG00000022580	INKA2	0.0000	0.0000	Up	0.0004	0.0113	Up			
ENSBTAG00000022715	-	0.0000	0.0000	Down						
ENSBTAG00000022779	OLFM4	0.0000	0.0000	Down	0.0000	0.0003	Down			
ENSBTAG00000023026	-	0.0000	0.0000	Up	0.0000	0.0000	Up			
ENSBTAG00000023411	-	0.0000	0.0000	Down	0.0000	0.0000	Down			
ENSBTAG00000024493	DHRS3	0.0000	0.0000	Down						
ENSBTAG00000024984	FSBP				0.0000	0.0018	Up			
ENSBTAG00000025071	TENM2	0.0006	0.0070	Down						
ENSBTAG00000025441	HSPA1A				0.0000	0.0000	Down			
ENSBTAG00000025458	DPF3							0.0010	0.0338	Down
ENSBTAG00000025597	GRIN3B	0.0010	0.0107	Up				0.0003	0.0149	Down
ENSBTAG00000025659	ZNF618	0.0000	0.0000	Down						
ENSBTAG00000025755	RNF212B	0.0003	0.0034	Up						
ENSBTAG00000026326	SPN	0.0001	0.0014	Up						
ENSBTAG00000026501	MGC127055	0.0006	0.0067	Up						
ENSBTAG00000026704	CENPW	0.0000	0.0000	Up						
ENSBTAG00000026809	-							0.0007	0.0255	Down
ENSBTAG00000027134	DYNC1I1	0.0000	0.0000	Up						
ENSBTAG00000027279	-	0.0022	0.0211	Up						
ENSBTAG00000027562	C15H11orf94	0.0007	0.0083	Down						

ENSBTAG00000029897	MIR21	0.0000	0.0000	Down						
ENSBTAG00000029920	MIR25	0.0030	0.0273	Down						
ENSBTAG00000029956	MIR491				0.0004	0.0106	Down			
ENSBTAG00000029970	bta-mir-196b				0.0024	0.0404	Down			
ENSBTAG00000030071	bta-mir-24-2				0.0002	0.0060	Down			
ENSBTAG00000030173	QRICH2	0.0001	0.0018	Down	0.0001	0.0025	Down			
ENSBTAG00000030227	PCDHA13				0.0008	0.0179	Down			
ENSBTAG00000030259	RASGRF2	0.0000	0.0000	Down						
ENSBTAG00000030367	RNF186				0.0013	0.0260	Down			
ENSBTAG00000030384	SURF1	0.0000	0.0000	Up						
ENSBTAG00000030474	-	0.0000	0.0000	Down						
ENSBTAG00000030474	-							0.0000	0.0007	Up
ENSBTAG00000030517	SMIM33				0.0015	0.0288	Up			
ENSBTAG00000030705	DACT3							0.0001	0.0037	Down
ENSBTAG00000031287	TMEM158	0.0000	0.0006	Up						
ENSBTAG00000031383	SMIM24	0.0000	0.0000	Down				0.0000	0.0018	Up
ENSBTAG00000031395	C6H4orf48							0.0000	0.0015	Down
ENSBTAG00000031573	NMRK2				0.0009	0.0202	Down			
ENSBTAG00000031669	CTNNA2	0.0049	0.0405	Up						
ENSBTAG00000032152	TMPRSS6	0.0000	0.0000	Down						
ENSBTAG00000032281	-	0.0001	0.0014	Up						
ENSBTAG00000032450	-	0.0004	0.0047	Down						
ENSBTAG00000032613	SCG5	0.0000	0.0000	Down	0.0000	0.0000	Down	0.0016	0.0468	Up

ENSBTAG00000032656	CPN2				0.0002	0.0056	Down			
ENSBTAG00000032819	MUC20	0.0000	0.0000	Down				0.0000	0.0024	Up
ENSBTAG00000032821	SCEL	0.0000	0.0000	Up						
ENSBTAG00000033153	GRIK2	0.0000	0.0000	Up	0.0000	0.0000	Up			
ENSBTAG00000033545	EBD	0.0000	0.0000	Down						
ENSBTAG00000033580	-	0.0002	0.0030	Down						
ENSBTAG00000033690	BARD1	0.0000	0.0000	Up						
ENSBTAG00000033998	RFX8	0.0001	0.0011	Up	0.0028	0.0456	Up			
ENSBTAG00000034182	-	0.0003	0.0036	Up						
ENSBTAG00000034360	SERF1A	0.0000	0.0000	Up						
ENSBTAG00000034496	-	0.0000	0.0000	Down						
ENSBTAG00000034827	PDGFD	0.0031	0.0278	Up						
ENSBTAG00000034885	RGCC	0.0000	0.0000	Up						
ENSBTAG00000035544	CYP46A1				0.0002	0.0065	Down			
ENSBTAG00000035572	-	0.0052	0.0423	Up						
ENSBTAG00000036016	FAM111B	0.0000	0.0000	Down	0.0000	0.0000	Down			
ENSBTAG00000037429	HTR1B	0.0025	0.0236	Up						
ENSBTAG00000037558	GRO1	0.0000	0.0000	Down						
ENSBTAG00000037768	MMP3	0.0000	0.0002	Up				0.0000	0.0000	Down
ENSBTAG00000037778	CXCL3	0.0000	0.0000	Down						
ENSBTAG00000038180	SCN2A	0.0018	0.0178	Up						
ENSBTAG00000038337	MZB1	0.0014	0.0148	Down						
ENSBTAG00000038415	SLC6A12				0.0000	0.0001	Down			
ENSBTAG00000038495	DCLK1	0.0004	0.0047	Up						
ENSBTAG00000038534	ZNF446	0.0005	0.0063	Down						

ENSBTAG00000038889	RILP	0.0000	0.0000	Down						
ENSBTAG00000039028	PI3	0.0000	0.0000	Down						
ENSBTAG00000039154	-	0.0003	0.0034	Up						
ENSBTAG00000039160	VAV1	0.0000	0.0000	Down						
ENSBTAG00000039326	GCNT2	0.0001	0.0021	Down						
ENSBTAG00000039334	-				0.0001	0.0022	Up			
ENSBTAG00000039397	WNT3A	0.0003	0.0039	Up						
ENSBTAG00000039446	-	0.0000	0.0000	Down						
ENSBTAG00000039446	-				0.0000	0.0000	Down			
ENSBTAG00000039711	ZFP62	0.0003	0.0038	Down				0.0000	0.0031	Up
ENSBTAG00000039849	-							0.0004	0.0157	Down
ENSBTAG00000040042	GRIN2C				0.0005	0.0135	Down	0.0005	0.0195	Down
ENSBTAG00000040056	LTB4R2	0.0003	0.0038	Up						
ENSBTAG00000040226	C11H2orf81				0.0011	0.0227	Up			
ENSBTAG00000040461	-	0.0000	0.0001	Down						
ENSBTAG00000042357	SNORD21	0.0030	0.0274	Up						
ENSBTAG00000042504	-							0.0016	0.0468	Down
ENSBTAG00000042536	SNORA2C	0.0000	0.0001	Down	0.0019	0.0337	Down			
ENSBTAG00000042553	SNORD99	0.0019	0.0184	Down						
ENSBTAG00000042757	-	0.0059	0.0470	Down						
ENSBTAG00000042757	-				0.0004	0.0108	Down			
ENSBTAG00000043023	U6	0.0013	0.0140	Up						
ENSBTAG00000043054	-	0.0049	0.0403	Up						
ENSBTAG00000043202	-	0.0003	0.0036	Down						

ENSBTAG00000043295	SNORA13							0.0009	0.0301	Down
ENSBTAG00000043315	SNORA61				0.0016	0.0306	Down			
ENSBTAG00000043412	SNORA71							0.0013	0.0391	Down
ENSBTAG00000044113	IZUMO2	0.0024	0.0225	Up						
ENSBTAG00000044175	CENPK				0.0000	0.0004	Up			
ENSBTAG00000044208	DUSP4				0.0000	0.0000	Down	0.0000	0.0006	Down
ENSBTAG00000044868	bta-mir-1843	0.0040	0.0344	Down						
ENSBTAG00000045270	bta-mir-2456	0.0053	0.0430	Up						
ENSBTAG00000045309	bta-mir-2443	0.0004	0.0055	Down						
ENSBTAG00000045482	U6	0.0003	0.0040	Up						
ENSBTAG00000045530	SCARNA2	0.0009	0.0100	Down						
ENSBTAG00000045567	PRR15	0.0000	0.0000	Down						
ENSBTAG00000045610	CACNA1I	0.0010	0.0112	Up						
ENSBTAG00000045625	NTN3	0.0005	0.0060	Down						
ENSBTAG00000045699	CTNNA3	0.0054	0.0434	Up						
ENSBTAG00000045746	-	0.0004	0.0047	Down						
ENSBTAG00000045746	-				0.0001	0.0035	Down			
ENSBTAG00000045767	LAT2							0.0009	0.0307	Up
ENSBTAG00000045925	CITED1	0.0058	0.0460	Up						
ENSBTAG00000046014	GPR146	0.0000	0.0000	Down						
ENSBTAG00000046148	-	0.0000	0.0005	Down						
ENSBTAG00000046158	CFB	0.0000	0.0000	Down	0.0000	0.0000	Down			

ENSBTAG00000046324	-	0.0000	0.0000	Down				0.0000	0.0000	Up
ENSBTAG00000046375	-	0.0000	0.0000	Down	0.0000	0.0000	Down			
ENSBTAG00000046409	EGR2	0.0007	0.0080	Up						
ENSBTAG00000046666	TTC9B				0.0000	0.0002	Down			
ENSBTAG00000046701	FRAT2							0.0001	0.0059	Down
ENSBTAG00000046900	GGT1	0.0000	0.0000	Down	0.0000	0.0000	Down			
ENSBTAG00000047231	TNNI1	0.0034	0.0302	Down						
ENSBTAG00000047326	EDA2R	0.0001	0.0015	Up	0.0001	0.0042	Up			
ENSBTAG00000047676	PRODH	0.0000	0.0000	Down						
ENSBTAG00000048029	MMP1							0.0000	0.0000	Down
ENSBTAG00000048086	-	0.0000	0.0002	Up						
ENSBTAG00000048138	XKR9	0.0002	0.0033	Up				0.0018	0.0497	Down
ENSBTAG00000048396	-	0.0000	0.0006	Down						
ENSBTAG00000048562	-	0.0004	0.0050	Down	0.0000	0.0000	Down			
ENSBTAG00000048660	C17orf113	0.0001	0.0014	Down						
ENSBTAG00000048676	U6				0.0010	0.0218	Up			
ENSBTAG00000048701	-	0.0000	0.0004	Down						
ENSBTAG00000048723	-	0.0049	0.0403	Up						
ENSBTAG00000048801	bta-mir-12038							0.0001	0.0038	Up
ENSBTAG00000048854	-	0.0009	0.0099	Up						
ENSBTAG00000048867	-	0.0003	0.0035	Down						
ENSBTAG00000049001	-	0.0009	0.0098	Up						
ENSBTAG00000049204	-	0.0012	0.0130	Up						
ENSBTAG00000049343	-	0.0001	0.0014	Up						

ENSBTAG00000049473	OPRL1	0.0007	0.0085	Up						
ENSBTAG00000049475	-	0.0027	0.0245	Up						
ENSBTAG00000049555	-				0.0014	0.0277	Down			
ENSBTAG00000049561	-	0.0026	0.0240	Down						
ENSBTAG00000049589	SAA3	0.0000	0.0000	Down	0.0000	0.0000	Down	0.0000	0.0000	Up
ENSBTAG00000049679	-	0.0025	0.0235	Down						
ENSBTAG00000049729	-							0.0004	0.0181	Down
ENSBTAG00000049747	-	0.0000	0.0001	Down	0.0012	0.0241	Down			
ENSBTAG00000049765	-	0.0000	0.0002	Up						
ENSBTAG00000049838	-	0.0000	0.0000	Up						
ENSBTAG00000049888	-	0.0002	0.0024	Down	0.0000	0.0010	Down			
ENSBTAG00000049893	-	0.0037	0.0322	Down						
ENSBTAG00000049916	Metazoa_ SRP	0.0028	0.0256	Down				0.0018	0.0488	Up
ENSBTAG00000049944	-				0.0009	0.0196	Down			
ENSBTAG00000050000	-	0.0000	0.0000	Down	0.0000	0.0001	Down	0.0000	0.0007	Up
ENSBTAG00000050072	-	0.0002	0.0025	Down						
ENSBTAG00000050189	-	0.0042	0.0357	Down						
ENSBTAG00000050269	-				0.0004	0.0106	Down			
ENSBTAG00000050279	-	0.0013	0.0140	Down						
ENSBTAG00000050388	KRBA2	0.0000	0.0004	Down						
ENSBTAG00000050398	-	0.0000	0.0000	Down						
ENSBTAG00000050486	-							0.0010	0.0315	Down
ENSBTAG00000050658	-	0.0032	0.0290	Up						
ENSBTAG00000050699	-	0.0006	0.0073	Up				0.0006	0.0239	Down

ENSBTAG00000050714	-	0.0039	0.0334	Down						
ENSBTAG00000050744	-	0.0007	0.0080	Up						
ENSBTAG00000050813	-							0.0014	0.0412	Down
ENSBTAG00000050877	-				0.0000	0.0009	Up			
ENSBTAG00000050968	-				0.0012	0.0241	Up			
ENSBTAG00000051001	-	0.0012	0.0128	Down						
ENSBTAG00000051094	-	0.0000	0.0001	Up						
ENSBTAG00000051099	-	0.0000	0.0006	Down	0.0008	0.0178	Down			
ENSBTAG00000051116	C6orf132	0.0000	0.0002	Up						
ENSBTAG00000051247	-				0.0002	0.0069	Up			
ENSBTAG00000051295	FXYD4	0.0056	0.0451	Down						
ENSBTAG00000051732	-	0.0001	0.0021	Down	0.0025	0.0414	Down			
ENSBTAG00000051780	RNASE12							0.0016	0.0452	Up
ENSBTAG00000051975	-	0.0000	0.0000	Up	0.0000	0.0001	Up			
ENSBTAG00000052053	-	0.0014	0.0143	Down						
ENSBTAG00000052100	-	0.0000	0.0000	Down						
ENSBTAG00000052223	-	0.0031	0.0278	Up						
ENSBTAG00000052443	-				0.0000	0.0008	Down			
ENSBTAG00000052508	SEM1	0.0000	0.0000	Up	0.0003	0.0080	Up			
ENSBTAG00000052611	-							0.0006	0.0240	Down
ENSBTAG00000052678	bta-mir-29c	0.0001	0.0012	Down						
ENSBTAG00000052686	-				0.0023	0.0391	Up			
ENSBTAG00000052693	-	0.0056	0.0446	Up						
ENSBTAG00000052696	-							0.0001	0.0079	Up

ENSBTAG00000052716	-	0.0000	0.0000	Up						
ENSBTAG00000052787	FAM71A	0.0002	0.0028	Up						
ENSBTAG00000052794	SSLP1							0.0003	0.0135	Down
ENSBTAG00000052846	-	0.0000	0.0000	Up						
ENSBTAG00000052866	-	0.0000	0.0000	Up	0.0000	0.0001	Up			
ENSBTAG00000052912	-	0.0000	0.0000	Up						
ENSBTAG00000053023	LRRC74B	0.0001	0.0010	Down						
ENSBTAG00000053028	-	0.0006	0.0069	Down						
ENSBTAG00000053057	-	0.0030	0.0272	Down						
ENSBTAG00000053163	-	0.0000	0.0000	Down						
ENSBTAG00000053202	-				0.0001	0.0036	Down			
ENSBTAG00000053240	-	0.0000	0.0000	Down	0.0012	0.0239	Down			
ENSBTAG00000053343	-	0.0000	0.0000	Down	0.0000	0.0005	Down			
ENSBTAG00000053383	-							0.0017	0.0478	Down
ENSBTAG00000053402	-				0.0006	0.0137	Up	0.0001	0.0067	Up
ENSBTAG00000053414	-				0.0000	0.0015	Up			
ENSBTAG00000053442	-	0.0002	0.0022	Down						
ENSBTAG00000053443	RUM1	0.0000	0.0000	Down	0.0001	0.0024	Down			
ENSBTAG00000053557	DEFB4A	0.0015	0.0151	Down						
ENSBTAG00000053603	-							0.0017	0.0476	Up
ENSBTAG00000053741	-	0.0000	0.0004	Down						
ENSBTAG00000053874	-	0.0047	0.0392	Down						
ENSBTAG00000053896	TOP1MT							0.0000	0.0005	Up
ENSBTAG00000053905	-	0.0003	0.0034	Down						
ENSBTAG00000053929	-				0.0007	0.0156	Down			

ENSBTAG00000054005	-							0.0017	0.0474	Down
ENSBTAG00000054045	-	0.0000	0.0000	Down						
ENSBTAG00000054057	NRG1	0.0000	0.0000	Up	0.0000	0.0002	Up			
ENSBTAG00000054065	-	0.0003	0.0035	Up						
ENSBTAG00000054218	IGFBP5	0.0000	0.0000	Down				0.0004	0.0159	Up
ENSBTAG00000054253	-	0.0000	0.0001	Up	0.0030	0.0484	Up			
ENSBTAG00000054278	MSAA3.2	0.0000	0.0000	Down	0.0002	0.0067	Down	0.0001	0.0036	Up
ENSBTAG00000054291	-				0.0018	0.0333	Up			
ENSBTAG00000054340	-				0.0013	0.0259	Up			
ENSBTAG00000054358	-				0.0015	0.0294	Up	0.0004	0.0178	Up
ENSBTAG00000054394	-	0.0007	0.0080	Down						
ENSBTAG00000054445	-	0.0016	0.0161	Down						
ENSBTAG00000054467	-				0.0017	0.0315	Up			
ENSBTAG00000054574	-	0.0000	0.0000	Up	0.0014	0.0271	Up			
ENSBTAG00000054644	-				0.0016	0.0302	Up			
ENSBTAG00000054661	bta-mir-2887-2							0.0001	0.0066	Down
ENSBTAG00000054793	-	0.0004	0.0055	Down						
ENSBTAG00000054993	TEX49	0.0013	0.0140	Up						
ENSBTAG00000055044	-	0.0056	0.0452	Up						
ENSBTAG00000055046	-							0.0001	0.0060	Down
ENSBTAG00000055223	-	0.0000	0.0000	Down						
ENSBTAG00000055237	-	0.0028	0.0256	Down						
ENSBTAG00000055238	-	0.0033	0.0296	Down						
ENSBTAG00000055282	CLDN2	0.0000	0.0000	Down	0.0000	0.0007	Down			
